# Multi-omics analysis reveals that natural hibernation is crucial for oocyte maturation in the female Chinese alligator

**DOI:** 10.1186/s12864-020-07187-5

**Published:** 2020-11-10

**Authors:** Jian-Qing Lin, Jun Yu, Hua Jiang, Yi Zhang, Qiu-Hong Wan, Sheng-Guo Fang

**Affiliations:** MOE Key Laboratory of Biosystems Homeostasis & Protection, State Conservation Centre for Gene Resources of Endangered Wildlife, College of Life Sciences, Zhejiang University, Hangzhou, 310058 People’s Republic of China

**Keywords:** Transcriptome, DNA methylation, miRNA, Epigenetics, Fertility, Hibernation environment

## Abstract

**Background:**

Hibernation in an appropriate environment not only is important for the survival of hibernators in winter, but also is crucial for breeding in the following season for many hibernating species. However, the genetic and epigenetic mechanism underlying this process remain unclear. In the current study, we performed an integrative multi-omics analysis of gonads collected from Chinese alligators that overwintered in wild cave and artificial warmroom to explore transcriptomic and epigenomic alternations in these organs.

**Results:**

The data revealed that in the breeding season, female alligators were more strongly affected in terms of gene expression than males by non-hibernation because of overwintering in a warm room, especially for genes related to oocyte maturation, and this effect commenced in winter with the downregulation of *STAR*, which is the rate limiting factor of steroid biosynthesis. Further, miRNAs were found to play essential roles in this negative effect of overwintering in the warm room on hibernation. The upregulated miRNAs likely were responsible for the suppression of oocyte maturation in the breeding season. Finally, DNA methylome changes, especially hypomethylation, were found to play an important role in the alterations in ovarian function-related gene expression induced by non-hibernation.

**Conclusions:**

Our study revealed the crucial role of hibernation quality for oocyte maturation in the Chinese alligator and the underlying genetic and epigenetic mechanisms, and highlights the importance of habitat, and especially, the overwintering site, in the conservation of not only the Chinese alligator, but also other endangered hibernators.

**Supplementary Information:**

The online version contains supplementary material available at 10.1186/s12864-020-07187-5.

## Background

Hibernation is an adaptive strategy adopted by many animals to survive the cold and foodless winter [1]. During winter, hibernators stay in their refuges and suppress their metabolic rates to substantially save energy, water, and oxygen [2]. The underlying molecular mechanisms vary between endotherms and ectotherms [3, 4]. Increasing evidence gathered since mid-last century suggests that there is a close correlation between the overwintering patterns and reproductive cycles of many mammals [5–7], birds [8], reptiles [9], amphibians [10], and invertebrates [11]. However, the importance of hibernation in gonadal development and reproduction remains controversial. For example, hibernation is crucial for oviposition and fertilization in the mountain yellow-legged frog (*Rana muscosa*) [10]. In the boreal toad (*Bufo boreas boreas*), hibernation would improve the breeding success in males, but not females [12]. However, a recent study on the Wyoming toad (*Anaxyrus baxteri*) revealed that hibernation is not essential for optimal sperm metrics in this species [13]. Thus, the relation between hibernation and reproduction varies among species. Further, our understanding of the molecular mechanisms underlying the effects of hibernation on reproduction is limited.

The Chinese alligator is a critically endangered freshwater crocodilian that once was widely distributed in China. However, because of climate change, hunting practices, and habitat destruction, its natural habitat is currently restricted to the lower Yangtze River, and the majority of animals live in captivity in two nature reserves in the Zhejiang and Anhui provinces of China. Chinese alligators stop eating and go into hibernation when the air temperature decreases in late October, and they emerge in late March as the temperature rises, and mate in late May [9, 14]. Hibernation quality has a crucial impact on their health and reproduction, and alligators made to overwinter in a warm room do not hibernate. When Chinese alligators move to a warmer area or overwinter in the discomfort of an artificial environment, they become less fertile [9, 15–17]. Thus, the Chinese alligator can serve as an ideal model to study the role of hibernation in reproduction, and the underlying molecular mechanisms.

Several genes involved in ovarian development and breeding activity, including *ESR1* (encoding estrogen receptor α) [18], *FSH* and *FSHR* (encoding follicle-stimulating hormone and its receptor) [19, 20], and *Kiss1* and *Kiss1R* (encoding Kisspeptin1 and its receptor) [21], are upregulated in the ovaries and several other tissues during the breeding season as compared to hibernation in the Chinese alligator. These results indicate that these genes may play a role in ovarian development and breeding activity after waking from hibernation; however, current knowledge is limited to a limited number of genes. Seasonal transcriptome changes in non-gonad tissues have been investigated in the Chinese alligator [4, 22] and other hibernators [3, 23–28]. We reasoned it would be interesting to use mRNA sequencing (mRNA-seq) to characterize genome-wide regulatory networks underlying the effects of hibernation on breeding success.

DNA methylation and small RNAs respectively are pre- and post-transcriptional epigenetic regulatory mechanisms. In this study, we carried out mRNA-seq, small RNA sequencing (sRNA-seq), and bisulfte sequencing (BS-seq) analyses of gonads collected in different seasons from Chinese alligators that overwintered in different overwintering environments to explore transcriptomic and epigenomic alterations induced by lack of hibernation.

## Results

### Data summary

**Fig. 1:**
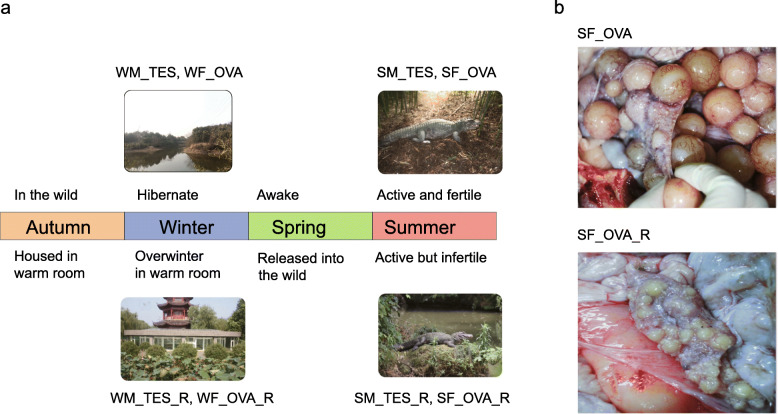
Chinese alligator gonadal tissue samples used in this study. **a** Annual life cycles of Chinese alligators in different overwintering environments and overview of the sample collection strategy used in this study. **b** The hibernated ovary (SF_OVA, above) and non-hibernation ovary (SF_OVA_R, below) collected in summer. The meaning of the letters in the sample names: W/S: Winter/Summer; M/F: Male/Female; TES/OVA: Testis/Ovary; R: overwinter in warm room. The photographs were taken by the authors of this paper

We carried out RNA-seq, sRNA-seq, and BS-seq analyses of ovary and testis samples collected in winter and summer from individual alligators that overwintered in the wild (hibernation) or in a warm room (non-hibernation) to gain insights into the molecular mechanisms underlying the effects of hibernation quality on gonadal development (Fig. 1a).

The eight stand-specific mRNA-seq libraries generated a total of 468.72 million reads, 419.72 million (89.55%) of which were uniquely mapped to the Chinese alligator reference genome [29]. mRNA-seq data analysis was based on the mapped reads, using a Chinese alligator gene annotation database containing 27,500 protein-coding genes [4]. sRNA-seq of the eight sRNA-seq libraries generated 100.63 million clean single-end reads, 85.63 million (85.10%) of which were 18–35 nt in length and were uniquely mapped to the Chinese alligator genome. sRNA-seq data analysis was based on the mapped reads and Chinese alligator sRNA annotation data generated in our previous study [4]. The eight BS-seq libraries generated 2955.58 million clean read pairs (669.17 Gb clean data), with an average depth of 18.38 per strand for each sample. On average, 92.65% of cytosines were covered by at least five unique reads in each sample. The bisulfite conversion rate was high in all libraries (99.51% on average).

### Transcriptional alterations in the maldeveloped ovary

**Fig. 2:**
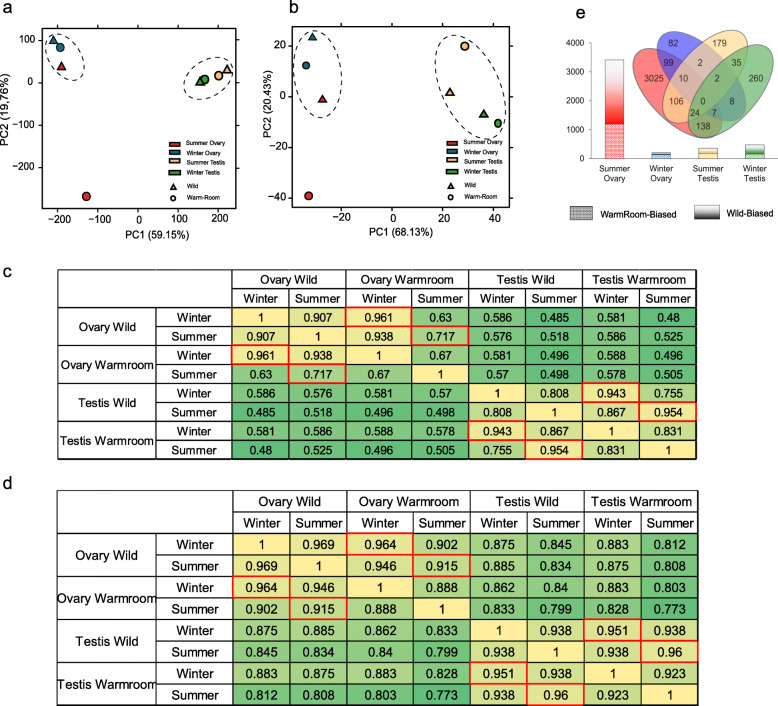
Analysis of gene and miRNA expression data of the gonads of Chinese alligators overwintering in different environments. **a**, **b**. Principal component analysis of mRNA (**a**) and miRNA (**b**) expression data of the gonads of Chinese alligators overwintering in different environments. **c**. Heatmap of Pearson correlation coefficients between each pair of mRNA-seq data of gonadal samples. **d**. Heatmap of Pearson correlation coefficients between each pair of sRNA-seq data of gonadal samples. **e**. Numbers of DEGs in gonads from animals overwintering in the wild (undergoing hibernation) and in those from animals overwintering in the warm room

To obtain an overview of the gonadal transcriptome changes in female and male alligators overwintering without hibernation, we carried out principal component analysis (PCA) of protein-coding gene and miRNA expression data from the eight gonadal samples. The results suggested that overwintering in the warm room led to ovarian maldevelopment, whereas testes were less affected. The four testis samples clustered together, and samples collected in the same season were located closer to each other in the PCA plot. The ovary sample collected in summer from the individual overwintering in the warm room (SF_OVA_R) was clearly separated from the three other ovary samples (Fig. 2a and b). Pearson correlation coefficients of mRNA-seq and sRNA-seq data for gonad samples were consistent with the PCA results (Fig. 2c and d). Comparison of gene expression levels in samples collected from animals overwintering in the warm room (WM_OVA_R, WM_TES_R, SF_OVA_R, and SF_TES_R) with those in corresponding samples of hibernating or hibernated animals (WF_OVA, WM_TES, SF_OVA, and SM_TES) revealed that there were substantially more differentially expressed genes (DEGs) in ovaries collected in summer (SF_OVA_R vs. SF_OVA) than in the other three pairs. Whereas there were only 474 and 358 DEGs in the testes collected in winter and summer and 210 DEGs in the ovaries collected in winter, there were 3412 DEGs in the ovaries collected in summer (Fig. 2e). These results suggested that hibernation is more important for ovarian than for testicular development and that gene/miRNA expression may substantially alter in the ovaries during the breeding season if the animal cannot hibernate in an appropriate environment. This is consistent with a previous report that overwintering in a man-made cave or in the warmer southern region of China led to maldevelopment of the gonads and breeding failure in the Chinese alligator [9]. Our results also suggest that this failure is likely caused by ovarian rather than testicular disorder.

**Fig. 3:**
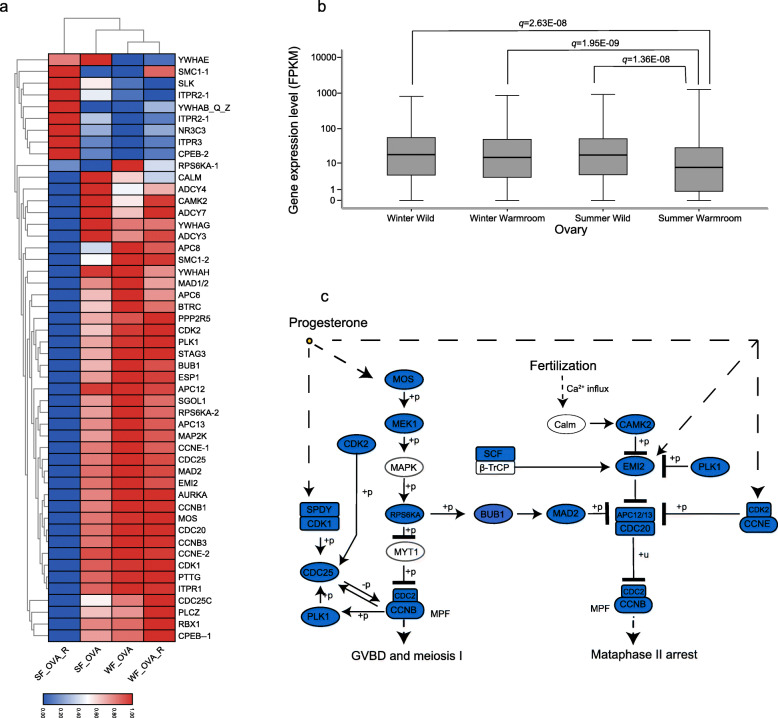
Gene expression related to oocyte maturation in the ovaries of Chinese alligators overwintering in different environments. **a**. Expression heatmap of DEGs involved in oocyte maturation. **b**. Box plots of the expression levels of genes involved in oocyte maturation pathway. **c**. Expression pattern of genes involved in oocyte maturation in SF_OVA_R samples

Next, we investigated KEGG pathway enrichment of DEGs that were downregulated in the SF_OVA_R sample as compared to the three other ovary samples. ‘Cell cycle’ and ‘oocyte meiosis’ were enriched in DEGs suppressed in the SF_OVA_R sample (q < 0.1) (Additional file 1: Table S1), suggesting that the breeding failure caused by overwintering in an inappropriate environment is due to suppression of oocyte meiosis and maturation. Indeed, a larger number of genes in the oocyte meiosis pathway were significantly suppressed in the SF_OVA_R sample (Wilcoxon signed-rank test, q < 0.05) (Fig. 3a and b). In particular, maturation-promoting factor (MPF), the key driver of meiotic progression, was suppressed in the SF_OVA_R sample. Genes in the Mos-MEK1-ERK2, Plk1-Cdc25, Bub1-Mad1/2-APC/Cdc20, and Camk2-Emi2 pathways, through which progesterone and fertilization induce resumption of the two meiotic division cycles and oocyte maturation, were also downregulated (Fig. 3c). These results are consistent with our finding that the ovarian follicle development is depressed in SF_OVA_R compared with that in SF_OVA (Fig. 1b). Several genes crucial for fertility (*GDF9*, *PRLRB*, *ADAMTS1*, *RBP4*, and *NANOS1*) [30–34], ovarian development (*BMP2A* and *BMP2B*) [35], and steroid hormone biosynthesis (*STAR*, *CYP11A*, *HSD17B1*, *LHX9*, *WT1*, and *SF1*) [36, 37] were also suppressed in the SF_OVA_R sample (Additional file 2: Figure S1). Interestingly, several male sex determination factors (including *MAP 3 K1*, *PTCH1*, *SOX9*, *FGFR2A*, and *AR*) [36, 37] were upregulated in this sample, suggesting that repression of the male gonadal developmental pathway was disturbed (Additional file 2: Figure S1). Together, these data indicated that gene expression in the ovaries is severely distorted in the breeding period when the Chinese alligator cannot hibernate in an appropriate environment.

Although females overwintering in the warm room did not show significant transcriptome alterations in the ovaries until the breeding period, changes in the ovaries had been triggered in winter. *STAR*, which encodes the rate-limiting enzyme steroidogenic acute regulatory protein, was downregulated in the ovaries of alligators overwintering in the warm room (WF_OVA_R) when compared with the levels in alligators hibernating in the wild (WF_OVA) (Additional file 2: Figure S1a and c). In contrast, *FGFR2A*, which regulates proliferation and Sertoli cell differentiation during testicular development, was upregulated in the WF_OVA_R sample, suggesting that the higher temperature in the warm room revoked the repression of male gonadal development during winter (Additional file 2: Figure S1a and c).

### MiRNA transcription alterations in the maldeveloped ovary

**Fig. 4:**
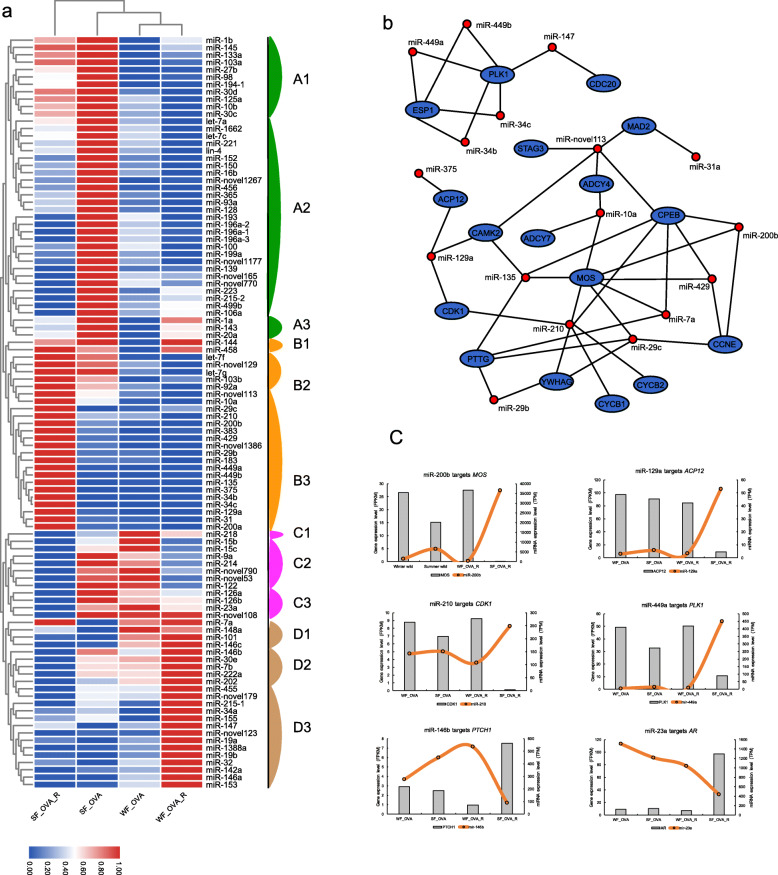
miRNA expression patterns in ovary samples of Chinese alligators overwintering in different environments. **a**. Expression heatmap of DEmiRs in the ovary samples. **b**. Interaction network showing regulatory effects of miRNAs on the downregulation of genes involved in oocyte maturation in the breeding season. **c**. Expression patterns of some genes involved in oocyte maturation and their putative regulatory miRNAs

To explore the roles of miRNAs in non-hibernation-induced infertility in the Chinese alligator, we constructed a heat map of the expression levels of differentially expressed miRNAs (DEmiRs) among the four ovary samples, and we classified all 96 DEmiRs into 12 groups based on their expression pattern (Fig. 4a). MiRNA expression clustering revealed a clear separation of the SF_OVA_R sample from the other three ovary samples, which was consistent with the PCA results (Fig. 2b). The expression patterns of these miRNAs suggested their potential involvement in ovarian development and function. For example, in the breeding season, miRNAs in group A2 were enhanced (e.g. miR-128 and let-7c) in the hibernated individual, but not in the individual that overwintered in warm room, suggesting that they may play crucial roles during the breeding season in the Chinese alligator and that overwintering in inappropriate environment inhibits their upregulation. MiRNAs in group C2 (e.g. miR-9a and miR-790) may be critical for ovarian development and function in both seasons as they their expression levels remained high in hibernating/hibernated animals, but decreased in individuals that spent their winter in the warm room. In contrast, miRNAs in groups B3 (e.g. miR-449a and miR-135) and D3 (e.g. miR-32 and miR-153) were upregulated during summer and winter respectively in the individuals overwintering in warmroom, suggesting their potential harmful impact on ovarian development and function.

To investigate the roles of miRNAs in gene expression regulation in non-hibernation-induced infertility in the Chinese alligator further, we predicted the targets of each miRNA and generated a regulatory network of their involvement in oocyte maturation (Fig. 4b). The results showed that most of the suppressed genes related to oocyte maturation were targeted by miRNAs (Fig. 4b) and, as expected, most of these miRNAs belonged to group B3 (Fig. 4a). For example, while miR-200b was upregulated, transcription of its target *MOS* was substantially supressed in the SF_OVA_R sample. Similar patterns were observed for other miRNA–gene pairs, including miR-129a–*ACP12*, miR-210–*CDK1*, and miR-449a–*PLK1* (Fig. 4c). These results suggest that oocyte maturation is actively repressed in the breeding season, at least in part, via miRNAs when the Chinese alligator did not hibernate in a suitable environment. In addition, the downregulation of miRNAs targeting male sex-determining genes (such as *PTCH1* and *AR*) likely results in the upregulation of these genes’ expression, which disturbs ovarian development in the breeding season (Fig. 4c). These results suggested that miRNAs play essential roles in ovarian maldevelopment induced by overwintering in an artificial environment.

### DNA methylome changes in the maldeveloped ovary

**Fig. 5:**
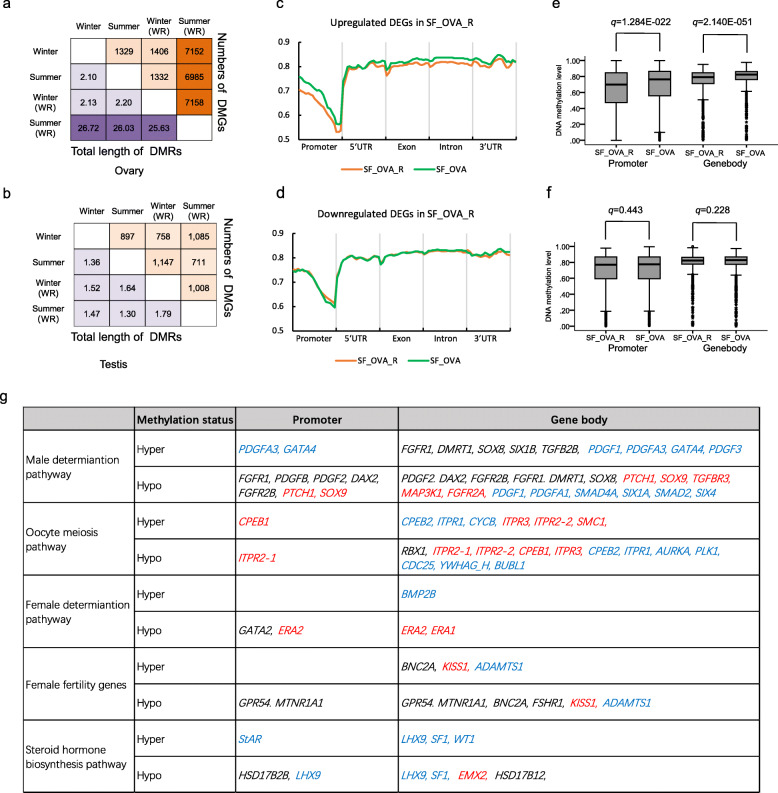
DNA methylation patterns in the gonads of Chinese alligators overwintering in different environments. **a**. Total lengths of DMRs and numbers of DMGs in ovary samples. **b**. Total lengths of DMRs and number of DMGs in testis samples. **c**, **d**. Distributions of methylation levels in different gene elements of upregulated (**c**) and downregulated DEGs (**d**) in SF_OVA_R samples. **e**, **f.** Box plots of methylation levels in promoters and gene bodies of upregulated (**e)** and downregulated DEGs in SF_OVA_R samples (**f**). **g**. DMGs involved in sex differentiation, fertility, and oocyte maturation, and expression change in the breeding season after overwintering in the warm room. Red: up-regulated DEG; Blue: down-regulated DEG; Black: not DEG.

To study DNA methylation changes in ovarian maldevelopment of the Chinese alligator, we compared the pairs of ovary samples and identified differentially methylated regions (DMRs) in the genomes. We found that an average of 26.13 Mb (1.186% of the genome) were DMRs between the SF_OVA_R sample and the three other ovary samples, and an average of 2.14 Mb (0.097% of the genome) were DMRs between each pair of the three other ovary samples (Fig. 5a), whereas only 1.51 Mb (0.069% of the genome) were DMRs among the four testis samples (Fig. 5b). These patterns were consistent with the transcriptome data (Fig. 2a, b).

We next focused on DMRs between the two ovary samples collected in summer to explore the epigenetic mechanism underlying ovarian maldevelopment. Compared with the ovary sample of the hibernated alligator (SF_OVA), there were 26.03 Mb DMRs in the ovary sample of the non-hibernated alligator collected (SF_OVA_R), including 20.48 Mb hypo-DMRs (corresponding to 5679 differently methylated genes, DMGs) and 5.54 Mb hyper-DMRs (corresponding to 2304 DMGs). The overlap of the DEGs and DMRs revealed that DMGs were likely to be DEGs (Fisher exact test, q = 1.079E-148) (Additional file 3: Fig S2a and b). In the SF_OVA_R sample, genes with hypo-DMRs in their promoters or/and gene bodies were more likely to be upregulated DEGs (Fisher’s exact test, q < 0.05) (Additional file 3: Fig. S2c and d), whereas only genes with hyper-DMRs in their promoter tended to be downregulated (Fisher’s exact test, q < 0.05) and those with hyper-DMRs in their gene body did not show significant trends (Fisher’s exact test, q = 0.203) (Additional file 3: Fig. S2e and f). In addition, the average DNA methylation levels across gene elements of upregulated DEGs were lower in SF_OVA_R than in the other samples (paired-samples T test, q < 0.05) (Fig. 5c–f). These results suggested that DNA methylome changes, especially hypomethylation, play an important role in gene regulation in ovarian maldevelopment caused by non-hibernation in winter.

To investigate the regulatory role of DNA methylation in ovarian maldevelopment further, we compiled a list of 157 genes that likely play roles in oocyte meiosis, female fertility, and sex determination, and investigated their methylation status (Additional file 4: Table S2). In total, 69 of these genes (45.586%) displayed significantly differential methylation patterns between SF_OVA_R and SF_OVA (Fig. 5g and Additional file 4: Table S2). As expected, there were many more hypo- than hyper-DMGs in summer (38 vs 12), and 19 genes contained both hyper- and hypo-DMRs (Fig. 5g and Additional file 4: Table S2). Among the nine DEGs with DMRs in their promoters, seven genes showed opposite changes in expression and methylation alterations. For example, the male sex determination genes *SOX9* and *PTCH1*, upregulation of which likely contributed to ovarian maldevelopment, had hypo-methylated promoters in the SF_OVA_R sample. In contrast, *STAR* was repressed and had a hyper-DMR in its promoter. However, there were more numerous DMGs with DMRs in their gene body, and the correlations between their methylation and expression were very complex. For example, some of the genes related to oocyte meiosis and downregulated in the SF_OVA_R sample had hyper-DMRs in their gene body (*CYCB*), some had hypo-DMRs in their gene body (*AURKA*, *PLK1*, *CDC25*, *YWHAG_H* and *BUBL1*), and others had both (*CPEB2* and *ITPR1*) or none (Fig. 5g).

### Seasonal transcriptional and epigenetic alterations in the testes

As mentioned above, the overwintering environment did not have a significant influence on seasonal transcriptional and epigenetic alterations in the testes. KEGG pathway and GO term analyses revealed that KEGG pathways involved in primary metabolic processes, gene expression, and protein metabolic processes, including ‘ribosome’, ‘spliceosome’, ‘RNA polymerase’, and ‘pyrimidine metabolism’ (q < 0.05, Additional file 5: Table S3) and the GO terms ‘ribosome’, ‘translation’, ‘gene expression’, ‘primary metabolic process’, and ‘protein metabolic process’ were enriched in DEGs upregulated in the testis samples of hibernating animals (q < 0.05, Additional file 6: Table S4). These results were in contrast with gene expression patterns reported in other tissues, in which energy metabolism and functional gene expression were suppressed [4], suggesting that the testes of the Chinese alligator actively develop during winter in preparation for reproduction in the next summer, but this process is not effect by the quality of overwinter environment. *AMH* (encoding anti-Müllerian hormone), *MTNR1A* (encoding melatonin receptor 1A), and the steroid hormone biosynthesis genes *CYP11A*, *HSD3B*, and *CYP17* were upregulated in summer (Additional file 7: Figure S3), suggesting the important roles of anti-Müllerian hormone, melatonin, and steroid hormone in male breeding.

## Discussion

Hibernation quality is known to have a substantial impact on reproduction in the Chinese alligator [9, 15]. In this study, we found that not only the transcriptome, but also the miRNome and DNA methylome are more strongly affected in female Chinese alligators by an inappropriate hibernation environment than in male animals. DEGs, DEmiRs, and DMGs potentially involved in this process were identified to uncover the complex molecular mechanisms underlying the influence of hibernation on the breeding success of the Chinese alligator.

While hibernation quality is known to affect animal reproduction in various species, its effects seem to vary among species and the sexes [10, 12, 13]. In the testes of the Chinese alligator, the transcriptome and epigenome showed season-biased changes (e.g. in hormone biosynthetic genes), but were not obviously affected by the overwintering environment (Fig. 2 and Fig. 5b). However, in the ovary, mRNA and miRNA expression patterns as well as DNA methylation patterns were significantly altered in the breeding season following a non-hibernation winter in the warm room (Fig. 2 and Fig. 5a). These results suggest that the female alligator is more sensitive to the hibernation environment. In line herewith, it is believed that the low temperatures experienced during hibernation are an important environmental factor for oocyte maturation in frogs and other hibernating species [38]. In the Chinese toad (*Bufo Bufo gargarizans*), low temperatures during hibernation contribute to oocyte maturation competence via the accumulation of CDC2, an MPF component, in the oocytes, and the CDC2 level was significantly reduced in toads disturbed from hibernation as a result of high temperatures [39]. Our results are consistent with these findings in Anura, and in the Chinese alligator ovary, in addition to MPF components, the entire oocyte maturation pathway was suppressed when an appropriate overwinter environment was lacking.

The fact that miRNAs play key roles in gonadal development is supported by knockout/knockdown studies and expression studies in mammals [40, 41]. Knockout or knockdown of miRNA machinery genes (*Dicer*, *Ago2*, and *DGCR8*) in mice led to a global suppression of miRNA functions, suppression of oocyte maturation, and abnormal preimplantation development [42, 43]. In our study, miRNA expression profiling identified 96 miRNAs that were differentially expressed between the four ovary samples and that likely played roles in ovarian maldevelopment in non-hibernation (Fig. 4a). Some of these miRNAs have been reported to be related to ovarian function. For example, miR-126, miR-15b, miR-143, and miR-199a, which are abundant in the mammalian ovary and are predicted to target ovarian function-related genes, were supressed in the breeding season after overwintering in the warm room. In addition, numerous miRNAs involved in oestradiol inhibition [44] were upregulated during winter (groups D1–D3: miR-148a, miR-101, miR-146, miR-7b, miR-34a, miR-19, and miR-32) or summer (groups B1–B3: let-7 g, miR-144, miR-103b, miR-92a, miR-10a, miR-135, miR-129a, miR-31) in female alligators that overwintered in the warm room, suggesting that oestrogen hormone biosynthesis in the ovaries is suppressed in female alligators that missed hibernation. Consistent herewith, *STAR*, which encodes a rate-limiting enzyme in the steroid biosynthesis pathway, was significantly downregulated in winter in the non-hibernating female alligator.

DNA methylation is crucial in ovarian development in various species, including the honeybee (*Apis mellifera*) [45, 46], Japanese flounder (*Paralichthys olivaceus*) [47, 48], zebrafish (*Danio rerio*) [49] and sheep (*Ovis aries*) [50]. Further, it participates in diseases that are of ovarian origin and directly cause infertility [51]. We found numerous DMRs, especially hypomethylated regions, in DNA collected in summer from female animals that missed hibernation, and we identified several DMGs related to ovarian function. Moreover, as the DMGs were enriched in TF genes (Fisher’s exact test, *p* = 6.386E-021), and given the *trans*-regulatory role of methylation in hibernation adaption of non-gonadal tissue [4], we reason that other DEGs in the SF_OVA_R sample were *trans*-regulated by the differentially methylated TFs as shown in the transcription network in Additional file 8: Figure S4.

In face of the threats from climate change, hunting, and habitat destruction, the wild Chinese alligator population has fallen sharply. Therefore, it is imperative to establish a captive population and implement a captive-breeding program for population rejuvenation. However, as the quality of the hibernation environment has a great impact on oocyte maturation in this alligator, the captive environment, and especially, the potential hibernation sites, should be paid more attention to and should more closely reflect overwintering sites in their wild habitat. In addition, human disturbance should be avoided in the hibernation season.

## Conclusions

Overall, our data provide genetic and epigenetic insights into the crucial significance of natural hibernation for reproduction of the Chinese alligator, especially for female oocyte maturation, and are expected to facilitate the development of scientific programs for successful conservation of not only the Chinese alligator, but also other endangered hibernators.

## Methods

### Sample sources and DNA and RNA extraction

Chinese alligator genital tissues were provided by the Changxing Yinjiabian Chinese Alligator Nature Reserve (CCANR) (Fig. 1a). Winter testis (WM_TES) and ovary (WF_OVA) samples were collected from two adult hibernating Chinese alligators (one male and one female) in January 2015 (the coldest time in Changxing), and summer samples (SM_TES and SF_OVA) were collected from another two adult individuals (one male and one female) during the breeding season, in May 2015. According to the guidelines of the Animal Ethics Committee of Zhejiang University, these animals were euthanized under deep anaesthesia by injecting ketamine (5–10 mg/kg) into the tail muscle and then followed by blood-letting. We collected not only gonads but also several other tissues used in another study. Another four adult individuals (two males and two females) were housed in a glass warm room at the CCANR in October 2014, where the alligators keep active during winter. Testis (WM_TES_R) and ovary (WF_OVA_R) samples were surgically obtained from one male and one female under deep anaesthesia by injecting ketamine (5–10 mg/kg) in January 2015. The other two individuals were released into the wild in late March 2015, and genital tissues (SM_TES_R and SF_OVA_R) of these animals were collected in the breeding season. The warmroom-overwintering alligators were released to the Changxing Yinjiabian Chinese Alligator Nature Reserve after their wounds healed well. The gonads are located in the posterior part of the abdominal cavity, in front of the kidneys. The testes are about 14*3*2 cm^3^ in size. The mature ovary is about 20*6*3 cm^3^, with many golden yellow follicles on it; while the undeveloped or maldeveloped ovary is about 10*3*2 cm^3^, on which the follicles are much smaller (Fig. 1b).

All samples were immediately stored in liquid nitrogen until use. All the experimental procedures with Chinese alligator in this study had been given prior approval by the Animal Ethics Committee of Zhejiang University (ZJU2015–154-13) and the State Forestry Administration of China [Forest Conservation Permission Document (2014) 1545].

The gDNA used for BS-seq and RNA used for mRNA-seq were extracted from the samples using an AllPrep DNA/RNA Mini Kit (Qiagen, Hilden, Germany), according to the manufacturer’s instructions. Total RNA for sRNA-seq was extracted using a TRIzol RNA isolation kit (Invitrogen, Waltham, MA) according to the manufacturer’s instructions.

### Strand-specific cDNA library construction and sequencing

Strand-specific cDNA libraries were generated from 3 mg RNA per sample, using a NEBNext® Ultra™ Directional RNA Library Prep Kit for Illumina® (New England Biolabs, Ipswich, MA) according to the manufacturer’s instructions. The index-coded samples were clustered using a TruSeq PE Cluster Kit v3-cBot-HS on a cBot Cluster Generation System (Illumina, San Diego, CA). The libraries were sequenced on an Illumina HiSeq 2500 platform, generating 125-bp paired-end reads. Reads containing adapter or poly-N sequences, and low-quality reads were removed using in-house Perl scripts, and all subsequent analyses were based on the clean reads.

### sRNA library construction and sequencing

sRNA libraries were prepared from 3 mg RNA per sample, using a NEBNext® Multiplex Small RNA Library Prep Set for Illumina® (NEB) according to the manufacturer’s instructions. The index-coded samples were clustered using a TruSeq SR Cluster Kit v3-cBot-HS on a cBot Cluster Generation System. After cluster generation, the sRNA libraries were sequenced on an Illumina HiSeq 2500 platform, generating 50-bp single-end reads. Reads containing 5′ adapter or poly-N sequences, reads without 3′ adapter or the insert tag, and low-quality reads were removed using in-house Perl scripts, and all subsequent analyses were based on the clean reads.

### BS library construction and sequencing

The gDNA was spiked with non-methylated λ DNA fragments for bisulfite conversion quality control purposes. For each sample, 6 mg gDNA and 30 ng λ DNA were mixed and fragmented into 200–300 bp using sonication. After end repair and acetylation, barcodes with methylated cytosines were added to the fragmented DNA. DNA bisulfite conversion was carried out twice using an EZ DNA methylation-Gold™ Kit (Zymo Research, Irvine, CA). The DNA fragments were then amplified by PCR using KAPA Hifi HotStart Uracil+ ReadyMix (Kapa Biosystems, Wilmington, MA). The index-coded samples were clustered using a TruSeq PE Cluster Kit v3-cBot-HS on a cBot Cluster Generation System (Illumina) according to the manufacturer’s instructions. The BS library was sequenced on the Illumina HiSeq 2500 platform, generating 125-bp paired-end reads. Low-quality reads, reads containing adapter or poly-N sequences, and reads shorter than 36 nt following adapter removal were filtered out using in-house Perl scripts, and all subsequent analyses were based on the clean reads.

### RNA-seq data analysis

An index of the Chinese alligator reference genome was built using Bowtie2 [52], and the clean reads were mapped to the reference genome using TopHat v. 2.0.12 [53]. Reads mapped to each gene were counted using HTSeq v. 0.6.1 [54] and differential gene expression between sample pairs was analysed using the DEGseq R package v. 1.12.0 [55]. Genes with a false discovery rate (FDR) < 0.05 and a |log2fold-change| > 1 were considered DEGs. The number of fragments per kilobase of exon per million (FPKM) mapped fragments was calculated to estimate gene expression levels. Gene expression level heatmaps were constructed using TBtools [56]. The DESeq R package [57] was used to carry out PCA of mRNA-seq data of the gonad samples and for plot construction.

### sRNA-seq data analysis

Clean sRNA reads were mapped to the Chinese alligator genome reference sequence using Bowtie v. 2.2.3 [52] without any mismatch allowed. We then annotated the reference genome sequence using the Rfam database [58] and RepeatMaker [59]. Reads corresponding to rRNAs, tRNAs, snRNAs, snoRNAs, repeat sequences, exons, and introns were removed. miREvo v. 1.1 [60] and mirdeep2 [61] were used to predict miRNAs through exploration of the secondary structure, the Dicer cleavage site, and the minimum free energy of the reads. Predicted miRNAs were subjected to Rfam [58] for miRNA family analysis and identification of conserved miRNAs (found in at least one other species). In-house scripts were used to obtain miRNA counts. miRNA expression levels were normalized as the number of transcripts mapped to the miRNA per million transcripts (TPM). The DEGseq R package was used to analyse differential miRNA expression between paired samples [55]. miRNAs with an FDR < 0.05 and a |log2fold-change| > 1 were regarded DEmiRs. MiRanda, RNAhybrid, and PITA were used to predict target genes of the miRNAs [62–64]. Target genes approved by at least one software package were considered targets. The DESeq R package [57] was use to carry out PCA of sRNA-seq data of the gonad samples and for plot construction.

### BS-seq data analysis

BS-seq reads were aligned to the Chinese alligator reference genome using Bismark (v. 0.12.5) with default parameters [65]. The reference genome was transformed into fully BS-converted versions termed ‘T genome’ (C-to-T converted) and ‘A genome’ (G-to-A converted) and then indexed using Bowtie2 [52]. All cytosines of the BS-converted reads were transformed to thymines, and the reads were aligned to the ‘T genome’. All guanines of the BS-converted reads were transformed to adenosines and the reads were aligned to the ‘A genome’. The sequence reads that produced a unique best alignment from the two alignments (original Watson and Crick strand) were aligned back to the original reference genome to infer the methylation state of all cytosines in the sequence reads. Multiple reads mapped to the same genome regions were regarded clonal duplicates and were removed to avoid inaccuracy that might be caused by PCR amplification bias. The BS library conversion rate was estimated as the percentage of cytosines sequenced at cytosine positions in the λ-DNA reference genome.

To identify methylation sites, we modelled the sum mC of methylated counts as a binomial (Bin) random variable with methylation rate (r), as mC ~ Bin (mC + umC*r).

The methylation level of each cytosine site was quantified as the number of reads containing an mC at the site of interest divided by the total number of reads covering the cytosine site. The methylation level of a specific region was quantified as the average methylation level of all cytosine sites in this region.

DMRs between two samples were identified using swDMR [66]. As most of the mC were in the CG context and the methylation levels in non-CG contexts were very low, we focused solely on CG sites. The sliding window was set to 1000 bp with a step length of 100 bp. To ensure statistical power, only windows with at least 10 CG sites and a coverage of at least 5 in each of the two compared samples were considered. Fisher’s exact test was used and only windows with FDR-adjusted *P* < 0.05 and a greater than twofold methylation level change were considered DMRs. Genes containing DMRs in their putative promoter or/and gene body regions were regarded DMGs.

### GO and KEGG enrichment analyses

GO term and KEGG pathway enrichment analyses were carried out using the GOseq 2.12 package and the KOBAS package, respectively [67, 68]. GO terms and KEGG pathways with an FDR (q value) < 0.05 were regarded significantly enriched.

## Supplementary Information

**Additional file 1 Table S1** Most enriched KEGG pathways in DEGs between SF_OVA_R and other ovary samples.

**Additional file 2 Figure** S1 Expression patterns of genes involved in sex differentiation and fertility in ovary samples from Chinese alligators overwintering in different environments. **a.** Expression alterations of genes involved in sex differentiation and fertility in the gonads of female alligators overwintering in the warm room. **b.** Expression heatmap of DEGs involved in sex differentiation and fertility in the ovary samples. **c.** Expression patterns of *GDF9*, *PRLRB*, *BMP2B*, *SOX9*, *StAR*, and *FGFR2A* in the ovary samples.

**Additional file 3 Figure S2** Correlations between DEGs and DMGs in ovaries in the breeding season. **a.** Venn diagram of DEGs and DMGs. **b.** Venn diagram of up- and downregulated DEGs and hyper- and hypomethylated DMGs. **c, d.** Venn diagrams of up- and downregulated DEGs and hypomethylated DMGs with DMR in promoters (**c**) and gene bodies (**d**). **e, f.** Venn diagrams of up- and downregulated DEGs and hypermethylated DMGs with DMR in promoters (**e**) and gene bodies (**f**).

**Additional file 4 Table S2** DMGs and/or DEGs between SF_OVA_R and SF_OVA samples in sex determination, fertility, and oocyte meiosis pathways.

**Additional file 5 Table S3** The most enriched (FDR < 0.1) pathways of season-biased DEGs in the testis of Chinese alligator.

**Additional file 6 Table S4** The most enriched (FDR < 0.05) GO terms of season-biased DEGs in the testis of Chinese alligator.

**Additional file 7 Figure S3** Gene expression related to the androgen biosynthesis pathway in the testes. a. Androgen biosynthesis pathway. Protein names in orange ovals indicate enzymes encoded by genes that are upregulated in testes collected in summer. b. Expression levels of *AMH*, *MTNR1A*, *CYP11A*, *HSD3B*, and *CYP17* in the testes.

**Additional file 8 Figure S4** Regulation of DEGs and DMGs encoding transcription factors (TFs). a. Venn diagram of TFs and DMGs. b. Venn diagram of TFs, DEGs, and DMGs. c. Associations between TFs and key genes with ovary-related functions.

## Data Availability

The Chinese alligator reference genome is available from GenBank (assembly accession: GCA_000455745.1). The BS-Seq, mRNA-Seq and sRNA-Seq data generated in this work have been deposited in the NCBI SRA database under BioProject accession numbers PRJNA556094, PRJNA556093, and PRJNA556092, respectively.

## Abbreviations

CCANR: Changxing Yinjiabian Chinese alligator nature reserve
W/F: Winter/summer
M/F: Male/female
TES/OVA: Testis/Ovary
R: Overwinter in warm room
mRNA-Seq: mRNA sequencing
sRNA-seq: Small RNA sequencing
BS-Seq: Bisulfte sequencing
DEG: Differentially expressed gene
DEmiR: Differentially expressed miRNA
DMR: Differentially methylated region
DMG: Differently methylated gene
PCA: Principal component analysis
FDR: False discovery rate
FPKM: The number of fragments per kilobase of exon per million
TPM: The number of transcripts mapped to the miRNA per million transcripts
ESR1: Estrogen receptor α
FSH: Follicle-stimulating hormone
FSHR: Follicle-stimulating hormone receptor
Kiss1: Kisspeptin1
Kiss1R: Kisspeptin1 receptor
MPF: Maturation-promoting factor
STAR: steroidogenic acute regulatory protein
AMH: Anti-müllerian hormone
MTNR1A: Melatonin receptor 1A.
