# Supplementary material for: Multi-omics analysis reveals that natural hibernation is crucial for oocyte maturation in the female Chinese alligator
Source: BMC Genomics. 2020 Nov 10;21:774. doi: 10.1186/s12864-020-07187-5 (PMC7653761; doi:10.1186/s12864-020-07187-5)
Supplement: Supplementary file 1 — Additional file 1 Table S1 Most enriched KEGG pathways in DEGs between SF_OVA_R and other ovary samples. [file 12864_2020_7187_MOESM1_ESM.pdf]

Table S1. Most enriched KEGG pathways in DEGs between SF_OVA_R and other ovary samples.

Term	ID	DEG number	ckground numb	P-Value	Corrected P-Value
**SF_OVAvsSF_OVA_R_up**					
ECM-receptor interaction	ko04512	37	99	1.52E-07	4.22E-05
Focal adhesion	ko04510	54	205	3.41E-06	0.000471916
Cell cycle	ko04110	34	111	1.70E-05	0.00156789
PI3K-Akt signaling pathway	ko04151	65	305	0.00011783	0.008159761
p53 signaling pathway	ko04115	18	56	0.000979822	0.054282143
Pathways in cancer	ko05200	66	348	0.001570822	0.067884806
Amoebiasis	ko05146	29	120	0.00179656	0.067884806
Protein digestion and absorption	ko04974	23	87	0.001960572	0.067884806
AGE-RAGE signaling pathway in diabetic complications	ko04933	25	100	0.00244397	0.069810227
Small cell lung cancer	ko05222	21	79	0.002846632	0.069810227
Oocyte meiosis	ko04114	24	96	0.002947963	0.069810227
Endocytosis	ko04144	48	242	0.00302427	0.069810227
MicroRNAs in cancer	ko05206	29	127	0.003637167	0.077499633
Bladder cancer	ko05219	12	35	0.004300893	0.085096238
**SF_OVAvsSF_OVA_R_down**					
Ribosome	ko03010	46	82	1.08E-23	2.85E-21
**WF_OVA_RvsSF_OVA_R_up**					
Cell cycle	ko04110	43	111	5.59E-08	1.57E-05
Oocyte meiosis	ko04114	30	96	0.000143888	0.020216209
**WF_OVA_RvsSF_OVA_R_down**					
Ribosome	ko03010	50	82	5.71E-24	1.53E-21
Protein processing in endoplasmic reticulum	ko04141	24	126	0.00015971	0.021401177
**WF_OVAvsSF_OVA_R_up**					
Cell cycle	ko04110	46	111	4.11E-08	1.14E-05
Endocytosis	ko04144	63	242	8.79E-05	0.012218641
Ubiquitin mediated proteolysis	ko04120	33	109	0.000476274	0.044134757
Oocyte meiosis	ko04114	29	96	0.001044214	0.072572846
**WF_OVA_RvsSF_OVA_R_down**					
Ribosome	ko03010	48	82	8.01E-19	2.22E-16
Protein processing in endoplasmic reticulum	ko04141	28	126	0.000155621	0.021553487
