# Supplementary material for: Multi-omics analysis reveals that natural hibernation is crucial for oocyte maturation in the female Chinese alligator
Source: BMC Genomics. 2020 Nov 10;21:774. doi: 10.1186/s12864-020-07187-5 (PMC7653761; doi:10.1186/s12864-020-07187-5)

a

		Putative sex determination/differentiation genes or fertility genes
Summer	Downregualted in SF_OVA_R	*BMP2A, BMP2B, GDF9, PRLRB, ADAMTS1, RBP4, NANOS1, LHX9, WT1, SF1, CYP17, HSD17B1, StAR, PDGFA1, PDGF1, PDGFC1, VNN1A, TGFB2A, FOG2, GATA4, PDGF3, GADD45G, PDGFA3*
	Upregualted in SF_OVA_R	*ERA1, ERA2, FST, KISS-1, RIP140, EMX2, MAP3K1, PTCH1, SOX9, FGFR2A, AR*
Winter	Downregualted in WF_OVA_R	*StAR*
	Upregualted in WF_OVA_R	*FGFR2A, FGFR1*

b

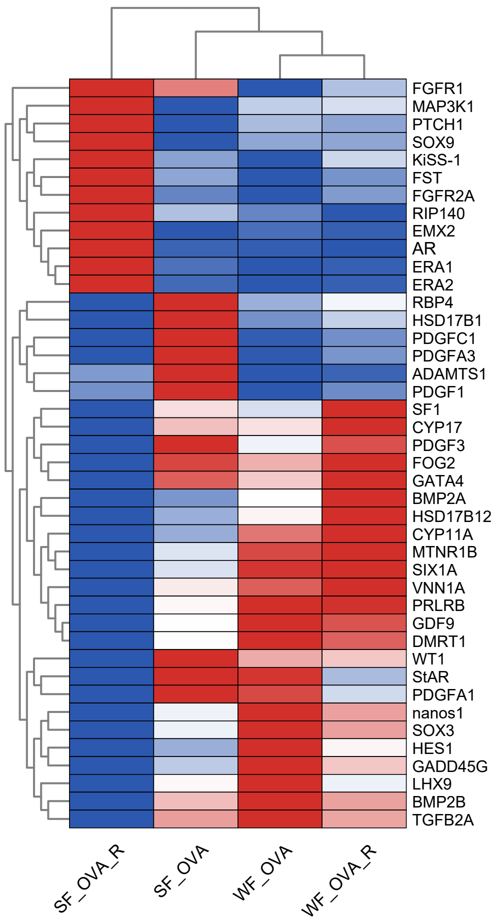

c

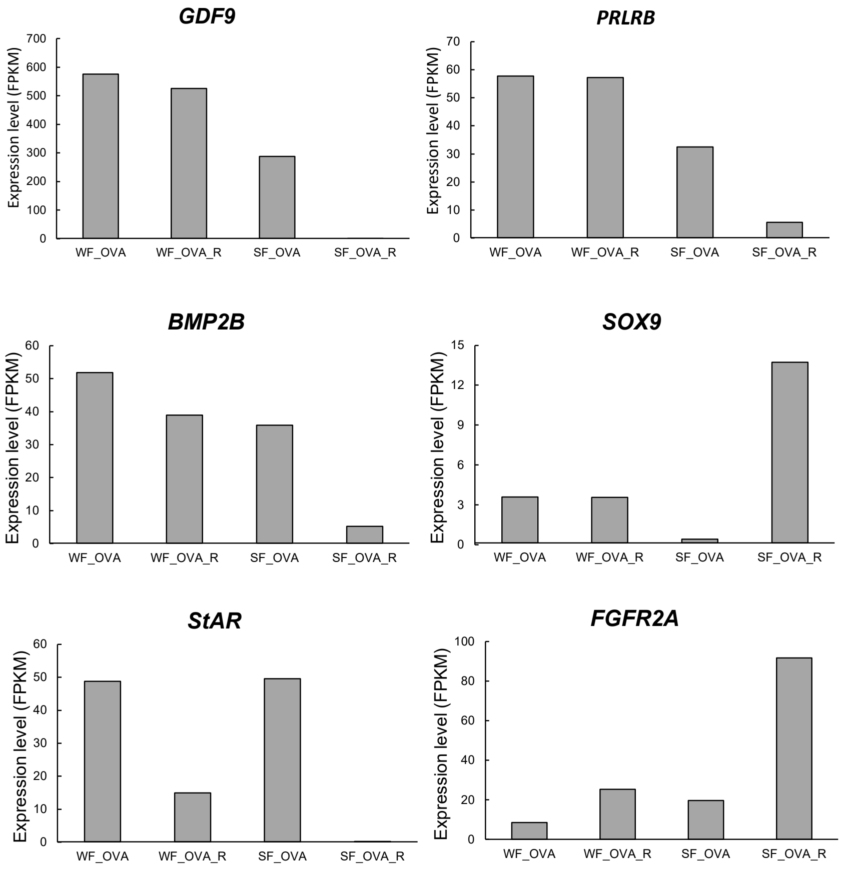

Supplement: Supplementary file 2 — Additional file 2 Figure S1 Expression patterns of genes involved in sex differentiation and fertility in ovary samples from Chinese alligators overwintering in different environments. a. Expression alterations of genes involved in sex differentiation and fertility in the gonads of female alligators overwintering in the warm room. b. Expression heatmap of DEGs involved in sex differentiation and fertility in the ovary samples. c. Expression patterns of GDF9, PRLRB, BMP2B, SOX9, StAR, and FGFR2A in the ovary samples. [file 12864_2020_7187_MOESM2_ESM.pdf]
