# Supplementary figures and images for: Multi-omics analysis reveals that natural hibernation is crucial for oocyte maturation in the female Chinese alligator
Source: BMC Genomics. 2020 Nov 10;21:774. doi: 10.1186/s12864-020-07187-5 (PMC7653761; doi:10.1186/s12864-020-07187-5)

a

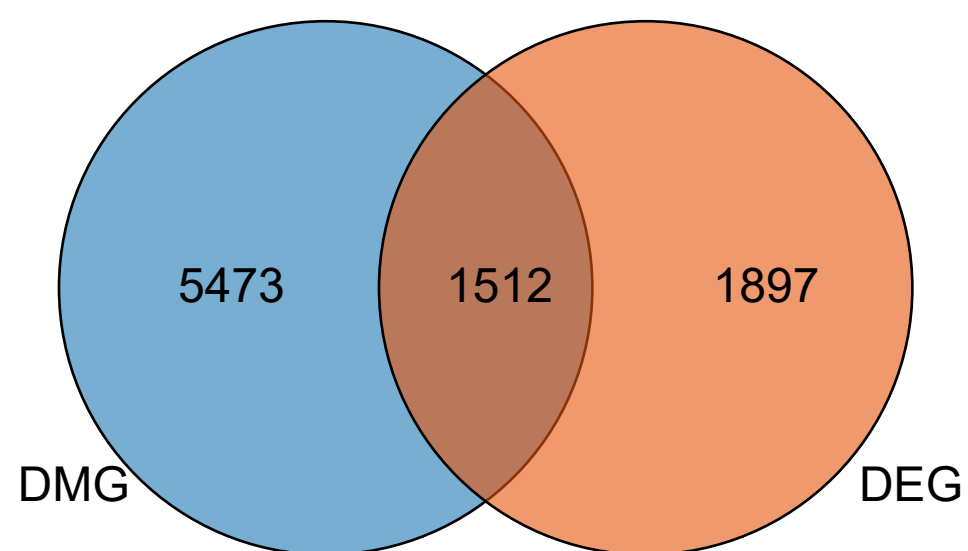

b

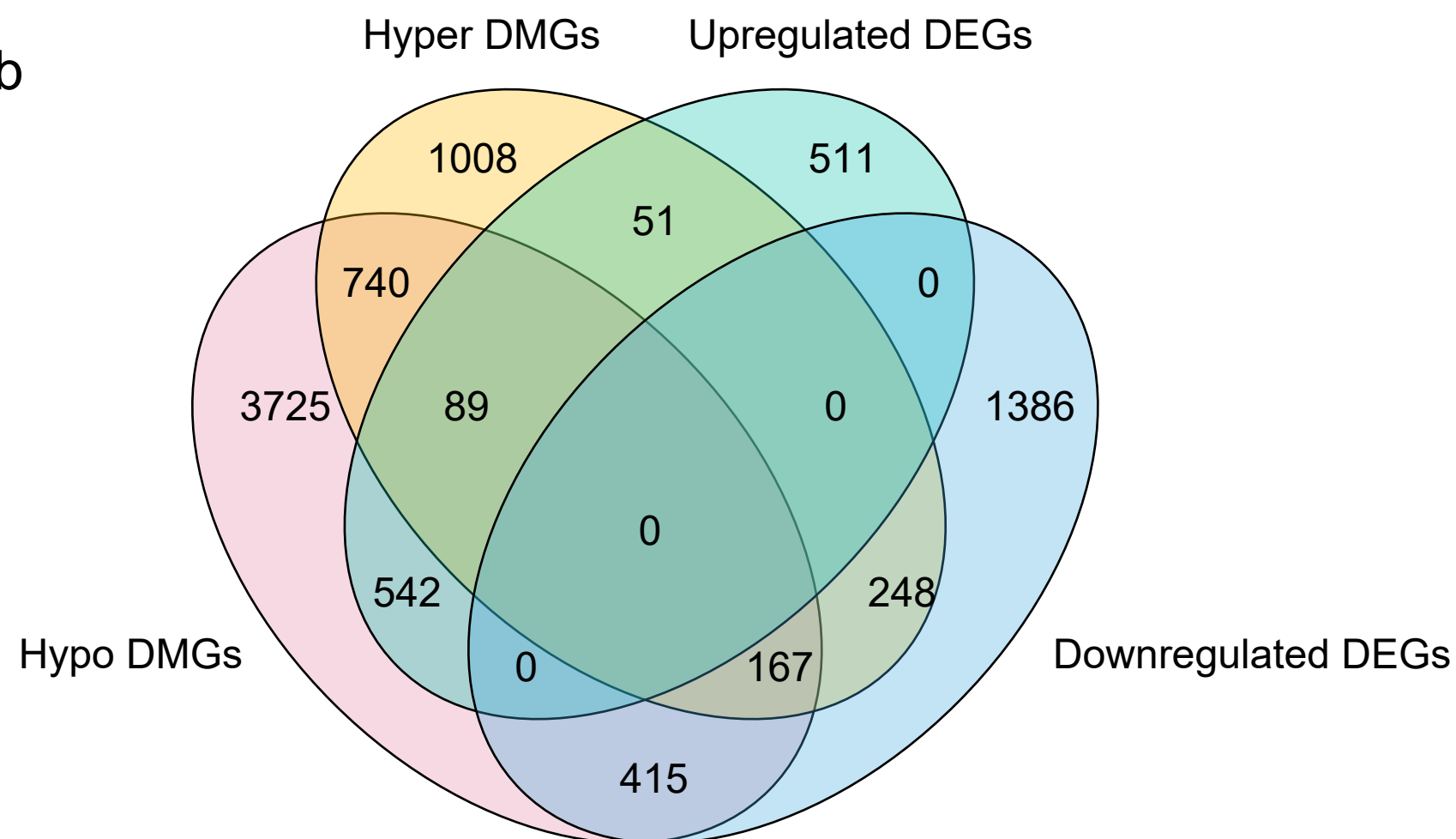

c

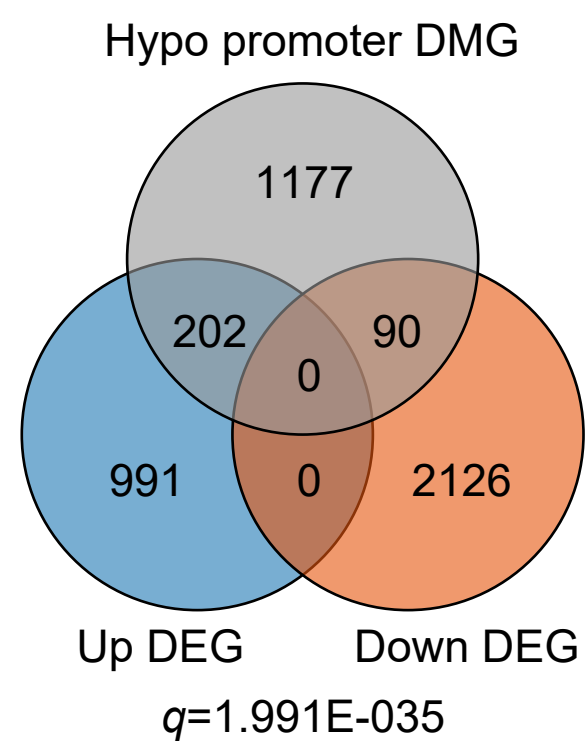

d

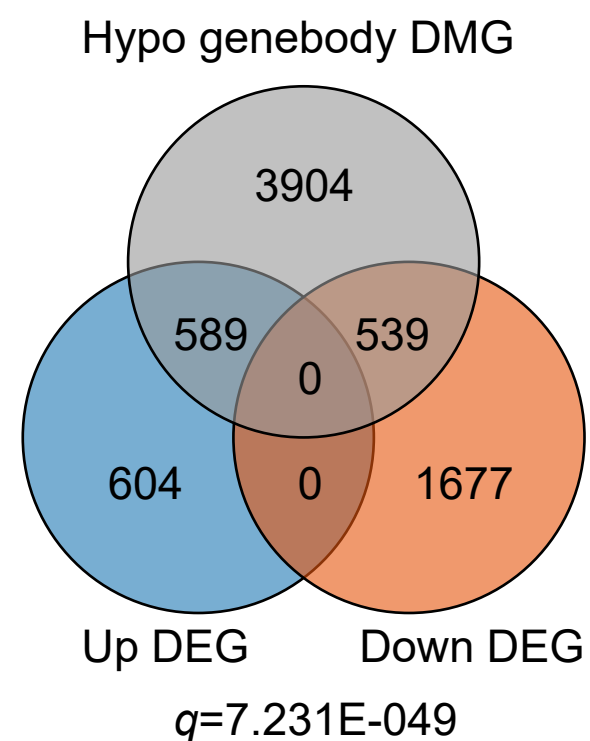

e

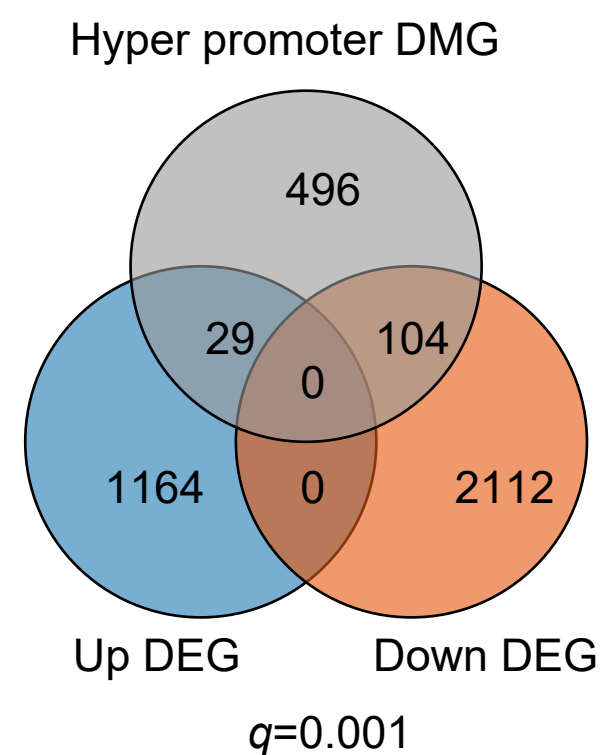

f

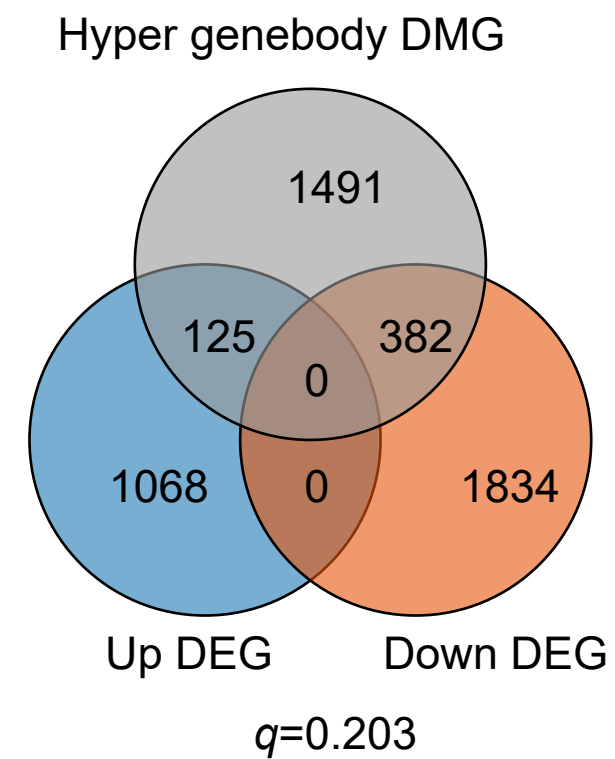

Supplement: Supplementary file 3 — Additional file 3 Figure S2 Correlations between DEGs and DMGs in ovaries in the breeding season. a. Venn diagram of DEGs and DMGs. b. Venn diagram of up- and downregulated DEGs and hyper- and hypomethylated DMGs. c, d. Venn diagrams of up- and downregulated DEGs and hypomethylated DMGs with DMR in promoters (c) and gene bodies (d). e, f. Venn diagrams of up- and downregulated DEGs and hypermethylated DMGs with DMR in promoters (e) and gene bodies (f). [file 12864_2020_7187_MOESM3_ESM.pdf]

a

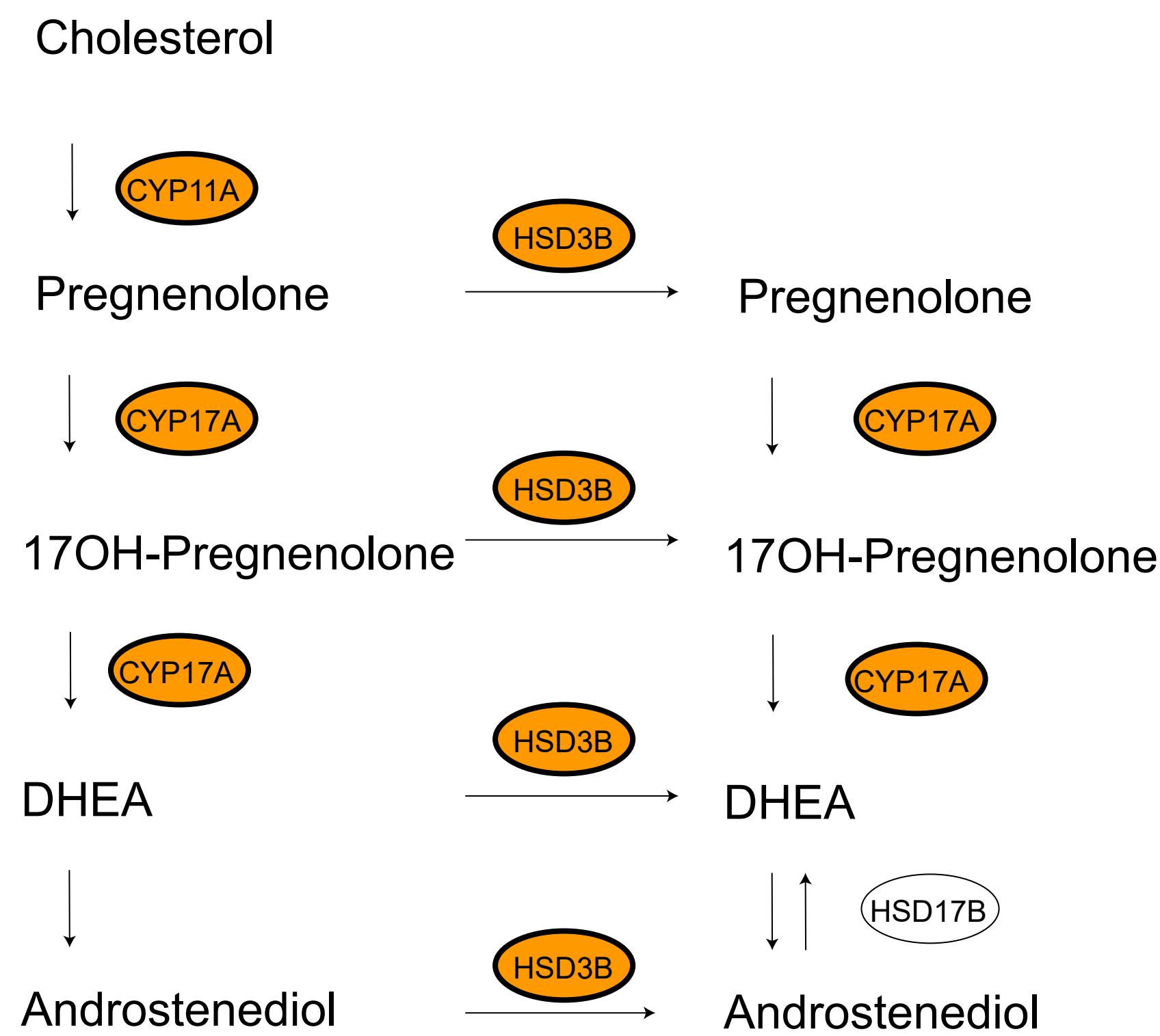

b

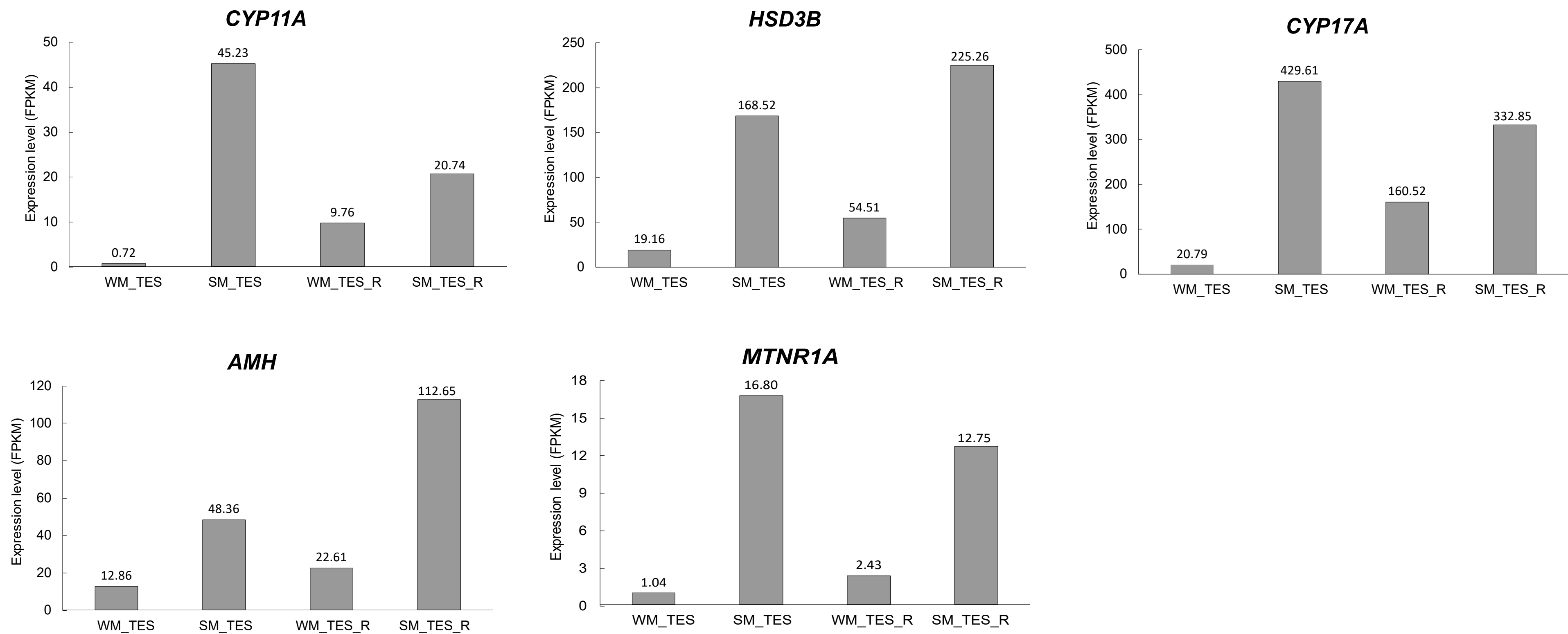

Supplement: Supplementary file 7 — Additional file 7 Figure S3 Gene expression related to the androgen biosynthesis pathway in the testes. a. Androgen biosynthesis pathway. Protein names in orange ovals indicate enzymes encoded by genes that are upregulated in testes collected in summer. b. Expression levels of AMH, MTNR1A, CYP11A, HSD3B, and CYP17 in the testes. [file 12864_2020_7187_MOESM7_ESM.pdf]

a

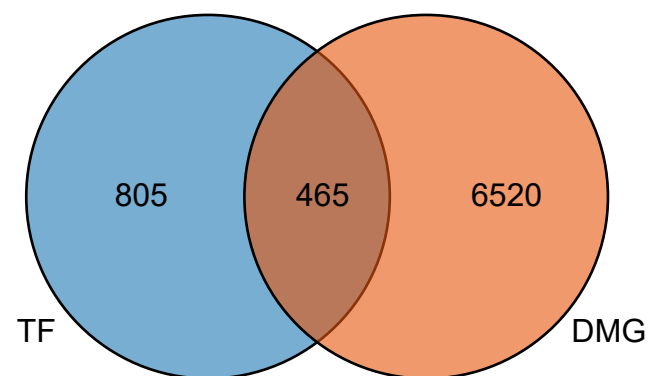

**b**

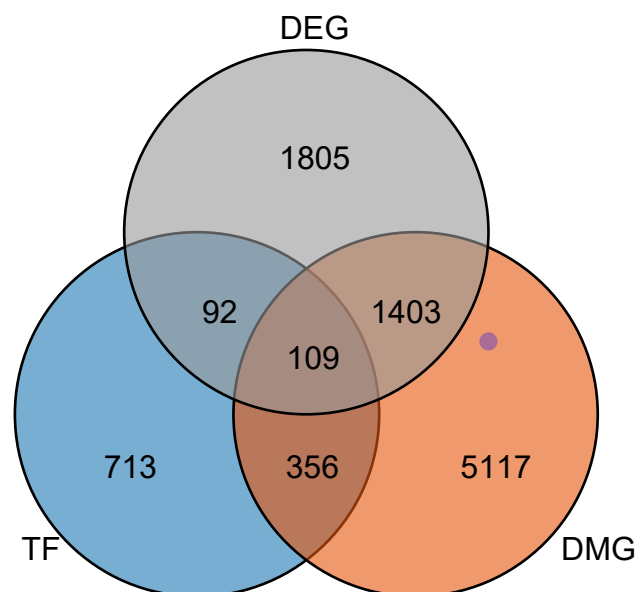

C

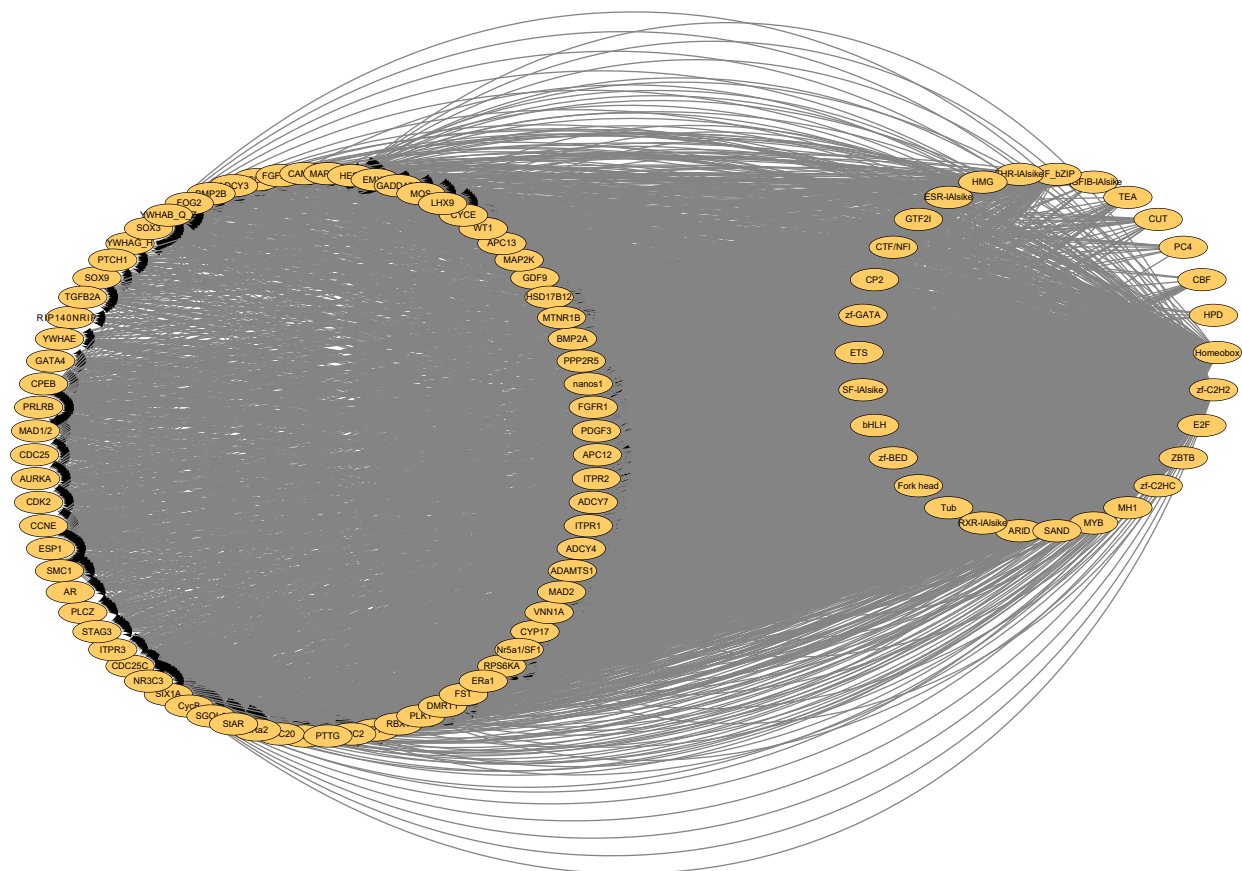

Supplement: Supplementary file 8 — Additional file 8 Figure S4 Regulation of DEGs and DMGs encoding transcription factors (TFs). a. Venn diagram of TFs and DMGs. b. Venn diagram of TFs, DEGs, and DMGs. c. Associations between TFs and key genes with ovary-related functions. [file 12864_2020_7187_MOESM8_ESM.pdf]
