# Supplementary material for: Multi-omics analysis reveals that natural hibernation is crucial for oocyte maturation in the female Chinese alligator
Source: BMC Genomics. 2020 Nov 10;21:774. doi: 10.1186/s12864-020-07187-5 (PMC7653761; doi:10.1186/s12864-020-07187-5)
Supplement: Supplementary file 4 — Additional file 4 Table S2 DMGs and/or DEGs between SF_OVA_R and SF_OVA samples in sex determination, fertility, and oocyte meiosis pathways. [file 12864_2020_7187_MOESM4_ESM.pdf]

Table S2. DMGs and/or DEGs between SF_OVA_R and SF_OVA samples in sex determination, fertility, and oocyte meiosis pathways

	Gene name	geneID	Differently expressed	Differently methylated
Female determiantion pathway	GATA2	Alsi24586	Not DEG	Hypo (promoter)
	ERa2	Alsi02441	Upregulated DEG	Hypo (exon, intron, promoter)
	ERa1	Alsi02440	Upregulated DEG	Hypo (exon, intron)
	BMP2B	Alsi05542	Downregulated DEG	Hyper (exon, intron)
	FST	Alsi19270	Upregulated DEG	Dot DMG
	BMP2A	Alsi00588	Downregulated DEG	Dot DMG
	WNT4A	Alsi03177	Not DEG	Dot DMG
	FOXL2	Alsi04067	Not DEG	Dot DMG
	Erb	Alsi05459	Not DEG	Dot DMG
	Rspo1	Alsi06967	Not DEG	Dot DMG
	BMP2C	Alsi07458	Not DEG	Dot DMG
	WNT5A	Alsi15079	Not DEG	Dot DMG
	CTNNB1	Alsi16947	Not DEG	Dot DMG
	WNT4B	Alsi17834	Not DEG	Dot DMG
Female fertility genes	GPR54	Alsi04020	Not DEG	Hypo (exon, intron, promoter)
	MTNR1A1	Alsi09629	Not DEG	Hypo (exon, intron, promoter)
	KiSS-1	Alsi04546	Upregulated DEG	Hypo (exon, intron); Hyper (exon, intron)
	BNC2A	Alsi26176	Not DEG	Hypo (exon, intron); Hyper (exon, intron)
	FSHR1	Alsi04466	Not DEG	Hypo (exon, intron)
	ADAMTS1	Alsi14668	Downregulated DEG	Hypo (exon, 5utr); Hyper (exon, intron)
	RIP140、NRIP1	Alsi17213	Upregulated DEG	Dot DMG
	GDF9	Alsi04981	Downregulated DEG	Dot DMG
	PRLRB	Alsi08086	Downregulated DEG	Dot DMG
	RBP4	Alsi17022	Downregulated DEG	Dot DMG
	nanos1	Alsi18184	Downregulated DEG	Dot DMG
	FSHR2	Alsi04467	Not DEG	Dot DMG
	FSH-β	Alsi07542	Not DEG	Dot DMG
	GnRHR2A	Alsi09727	Not DEG	Dot DMG
	GnRHR2B	Alsi15583	Not DEG	Dot DMG
	BMPR-1B	Alsi18123	Not DEG	Dot DMG
	MTNR1A2	Alsi18221	Not DEG	Dot DMG
	PRLR	Alsi18354	Not DEG	Dot DMG
	MTNR1B	Alsi19825	Not DEG	Dot DMG
	BMP15	Alsi20255	Not DEG	Dot DMG
	GnRHR2C	Alsi22688	Not DEG	Dot DMG
	BMP6	Alsi22940	Not DEG	Dot DMG
	Basonuclin-2B	Alsi26177	Not DEG	Dot DMG
Steroid hormone biosynthesis pathway	HSD17B2B	Alsi02771	Not DEG	Hypo (promoter)
	LHX9	Alsi14127	Downregulated DEG	Hypo (exon, intron, promoter); Hyper (exon, intron)
	Nr5a1/SF1	Alsi20150	Downregulated DEG	Hypo (exon, intron); Hyper (exon, intron)
	HSD17B12	Alsi08236	Not DEG	Hypo (exon, intron)
	EMX2	Alsi18186	Upregulated DEG	Hypo (exon, intron)
	StAR	Alsi27290	Downregulated DEG	Hyper (promoter)
	WT1	Alsi16145	Downregulated DEG	Hyper (exon, intron)
	CYP17	Alsi20333	Downregulated DEG	Dot DMG
	HSD17B1	Alsi23121	Downregulated DEG	Dot DMG
	HSD17B6A	Alsi00944	Not DEG	Dot DMG
	HSD17B6B	Alsi00945	Not DEG	Dot DMG
	HSD17B6C	Alsi00946	Not DEG	Dot DMG

	HSD17B6D	Alsi00947	Not DEG	Dot DMG
	CYP11A	Alsi01422	Not DEG	Dot DMG
	HSD17B2A	Alsi02770	Not DEG	Dot DMG
	HSD17B10	Alsi05190	Not DEG	Dot DMG
	HSD17B4A	Alsi08251	Not DEG	Dot DMG
	Cbx2	Alsi10378	Not DEG	Dot DMG
	CYP19A	Alsi11157	Not DEG	Dot DMG
	HSD17B6E	Alsi11168	Not DEG	Dot DMG
	HSD17B6F	Alsi11172	Not DEG	Dot DMG
	HSD17B4B	Alsi15752	Not DEG	Dot DMG
	HSD3B	Alsi15846	Not DEG	Dot DMG
Male determiantion pathyway	PDGFB	Alsi09917	Not DEG	Hypo (promoter)
	PDGF2	Alsi09175	Not DEG	Hypo (exon, intron, promoter)
	DAX2	Alsi15507	Not DEG	Hypo (exon, intron, promoter)
	FGFR2B	Alsi21317	Not DEG	Hypo (exon, intron, promoter)
	PTCH1	Alsi10845	Upregulated DEG	Hypo (exon, intron, promoter)
	SOX9	Alsi12198	Upregulated DEG	Hypo (exon, intron, promoter)
	FGFR1	Alsi27297	Not DEG	Hypo (exon, intron, 5utr, promoter); Hyper (exon, intron)
	DMRT1	Alsi12948	Not DEG	Hypo (exon, intron, 3utr); Hyper (exon, intron)
	PDGF1	Alsi02672	Downregulated DEG	Hypo (exon, intron); Hyper (exon, intron, 5utr)
	SOX8	Alsi03750	Not DEG	Hypo (exon, intron); Hyper (exon, intron)
	PDGFA1	Alsi01197	Downregulated DEG	Hypo (exon, intron)
	SMAD4A	Alsi02300	Not DEG	Hypo (exon, intron)
	SIX1A	Alsi04057	Not DEG	Hypo (exon, intron)
	SMAD2	Alsi06201	Not DEG	Hypo (exon, intron)
	SIX4	Alsi06593	Not DEG	Hypo (exon, intron)
	TGFBR3	Alsi09259	Not DEG	Hypo (exon, intron)
	MAP3K1	Alsi03214	Upregulated DEG	Hypo (exon, intron)
	FGFR2A	Alsi12452	Upregulated DEG	Hypo (exon, intron)
	AR	Alsi24145	Upregulated DEG	Hypo (exon, intron)
	PDGFA3	Alsi22609	Downregulated DEG	Hyper (exon, utr5, promoter)
	GATA4	Alsi15171	Downregulated DEG	Hyper (exon, intron, promoter)
	PDGF3	Alsi20201	Downregulated DEG	Hyper (exon, intron)
	SIX1B	Alsi04483	Not DEG	Hyper (exon, intron)
	TGFB2B	Alsi08442	Not DEG	Hyper (exon, intron)
	PDGFC1	Alsi03065	Downregulated DEG	Dot DMG
	VNN1A	Alsi04737	Downregulated DEG	Dot DMG
	TGFB2A	Alsi08441	Downregulated DEG	Dot DMG
	FOG2	Alsi09690	Downregulated DEG	Dot DMG
	GADD45G	Alsi21532	Downregulated DEG	Dot DMG
	CBLN4	Alsi02166	Not DEG	Dot DMG
	PDGFC2	Alsi03066	Not DEG	Dot DMG
	VNN1B	Alsi04738	Not DEG	Dot DMG
	SOX3	Alsi07043	Not DEG	Dot DMG
	HPGDS	Alsi09061	Not DEG	Dot DMG
	AMH	Alsi09101	Not DEG	Dot DMG
	SOX10	Alsi09946	Not DEG	Dot DMG
	SMAD4B	Alsi11147	Not DEG	Dot DMG
	SIX1C	Alsi17590	Not DEG	Dot DMG
	PDGFRB1	Alsi18631	Not DEG	Dot DMG
	PDGFRB2	Alsi18674	Not DEG	Dot DMG
	HES1	Alsi19126	Not DEG	Dot DMG

	PDGFA2	Alsi20609	Not DEG	Dot DMG
	HHAT	Alsi20694	Not DEG	Dot DMG
	KDM3A/JMJD1A	Alsi21323	Not DEG	Dot DMG
	MAP3K4	Alsi21820	Not DEG	Dot DMG
	FGF9	Alsi25321	Not DEG	Dot DMG
	SIX1D	Alsi26270	Not DEG	Dot DMG
oocyte meiosis pathway	ITPR2-1	Alsi22045	Upregulated DEG	Hypo (exon, intron, 5utr, promoter)
	CPEB1	Alsi26015	Upregulated DEG	Hypo (exon, intron); Hyper (promoter)
	CPEB2	Alsi14307	Downregulated DEG	Hypo (exon, intron); Hyper (exon, intron)
	ITPR1	Alsi27137	Downregulated DEG	Hypo (exon, intron); Hyper (exon, intron)
	ITPR3	Alsi25322	Upregulated DEG	Hypo (exon, intron); Hyper (exon, intron)
	AURKA	Alsi02168	Downregulated DEG	Hypo (exon, intron)
	PLK1	Alsi02207	Downregulated DEG	Hypo (exon, intron)
	CDC25	Alsi04090	Downregulated DEG	Hypo (exon, intron)
	YWHAG_H	Alsi05669	Downregulated DEG	Hypo (exon, intron)
	BUBL1	Alsi23916	Downregulated DEG	Hypo (exon, intron)
	RBX1	Alsi09906	Not DEG	Hypo (exon, intron)
	ITPR2-2	Alsi22052	Upregulated DEG	Hypo (exon, 5utr); Hyper (exon, 5utr)
	CYCB	Alsi20254	Downregulated DEG	Hyper (exon, intron)
	SMC1	Alsi05189	Upregulated DEG	Hyper (exon, intron)
	CDC25C	Alsi02364	Downregulated DEG	Dot DMG
	ESP1	Alsi06667	Downregulated DEG	Dot DMG
	CycB	Alsi07181	Downregulated DEG	Dot DMG
	CYCE	Alsi07936	Downregulated DEG	Dot DMG
	MAP2K	Alsi09737	Downregulated DEG	Dot DMG
	APC12	Alsi10642	Downregulated DEG	Dot DMG
	ADCY4	Alsi11431	Downregulated DEG	Dot DMG
	CCNE	Alsi11825	Downregulated DEG	Dot DMG
	PTTG	Alsi14334	Downregulated DEG	Dot DMG
	MAD1/2	Alsi17513	Downregulated DEG	Dot DMG
	YWHAG_H	Alsi17591	Downregulated DEG	Dot DMG
	MAD2	Alsi18467	Downregulated DEG	Dot DMG
	STAG3	Alsi18744	Downregulated DEG	Dot DMG
	CAMK2	Alsi19001	Downregulated DEG	Dot DMG
	ADCY3	Alsi20767	Downregulated DEG	Dot DMG
	CDC2	Alsi21664	Downregulated DEG	Dot DMG
	CDC20	Alsi23724	Downregulated DEG	Dot DMG
	RPS6KA	Alsi24503	Downregulated DEG	Dot DMG
	ADCY7	Alsi26352	Downregulated DEG	Dot DMG
	EMI2	Alsi26954	Downregulated DEG	Dot DMG
	MOS	Alsi27471	Downregulated DEG	Dot DMG
	NR3C3	Alsi08912	Upregulated DEG	Dot DMG
	YWHAB_Q_Z	Alsi25179	Upregulated DEG	Dot DMG
	CDK2	Alsi00930	Not DEG	Dot DMG
	APC13	Alsi01232	Not DEG	Dot DMG
	APC8	Alsi02366	Not DEG	Dot DMG
	PPP2R5	Alsi03183	Not DEG	Dot DMG
	CALM	Alsi04473	Not DEG	Dot DMG
	APC6	Alsi06036	Not DEG	Dot DMG
	BTRC	Alsi06894	Not DEG	Dot DMG
	YWHAE	Alsi09425	Not DEG	Dot DMG
	PLCZ	Alsi14434	Not DEG	Dot DMG

	SLK	Alsi20312	Not DEG	Dot DMG
	SGOL1	Alsi21166	Not DEG	Dot DMG
	RPS6KA	Alsi21546	Not DEG	Dot DMG
	SMC1	Alsi24095	Not DEG	Dot DMG
