# Supplementary material for: Multi-omics analysis reveals that natural hibernation is crucial for oocyte maturation in the female Chinese alligator
Source: BMC Genomics. 2020 Nov 10;21:774. doi: 10.1186/s12864-020-07187-5 (PMC7653761; doi:10.1186/s12864-020-07187-5)
Supplement: Supplementary file 5 — Additional file 5 Table S3 The most enriched (FDR < 0.1) pathways of season-biased DEGs in the testis of Chinese alligator. [file 12864_2020_7187_MOESM5_ESM.pdf]

Table S3. The most enriched (FDR<0.1) pathways of season-biased DEGs in the testis of Chinese alligator.

Term	ID	DEG number	Background number	P-Value	Corrected P-Value
**Wild, Winter-biased**					
Ribosome	ko03010	51	82	2.36E-35	5.56E-33
Spliceosome	ko03040	19	120	7.02E-06	0.000828102
RNA polymerase	ko03020	7	22	0.000168973	0.013292542
Pyrimidine metabolism	ko00240	11	73	0.000845532	0.049886375
Basal transcription factors	ko03022	7	32	0.001149942	0.054277252
RNA transport	ko03013	14	117	0.001393094	0.054795019
Ubiquitin mediated proteolysis	ko04120	13	109	0.002087979	0.070394729
mRNA surveillance pathway	ko03015	9	61	0.002781854	0.079285555
Ribosome biogenesis in eukaryotes	ko03008	8	50	0.003023602	0.079285555
Epstein-Barr virus infection	ko05169	17	176	0.003732283	0.088081888
**Wild, Summer-biased**					
/					
**Warmroom, Winter-biased**					
Ribosome	ko03010	25	82	3.16E-13	7.66E-11
Spliceosome	ko03040	21	120	1.28E-07	1.55E-05
Ribosome biogenesis in eukaryotes	ko03008	12	50	3.98E-06	0.000320672
RNA transport	ko03013	17	117	1.79E-05	0.00108568
Fanconi anemia pathway	ko03460	8	35	0.000218381	0.010569619
**Warmroom, Summer-biased**					
ECM-receptor interaction	ko04512	20	99	9.90E-05	0.025847539
Inositol phosphate metabolism	ko00562	13	57	0.000593489	0.058127703
Focal adhesion	ko04510	29	205	0.000814375	0.058127703
Amoebiasis	ko05146	20	120	0.000890846	0.058127703
Phosphatidylinositol signaling system	ko04070	16	87	0.001148184	0.0599352
