# Supplementary material for: Multi-omics analysis reveals that natural hibernation is crucial for oocyte maturation in the female Chinese alligator
Source: BMC Genomics. 2020 Nov 10;21:774. doi: 10.1186/s12864-020-07187-5 (PMC7653761; doi:10.1186/s12864-020-07187-5)
Supplement: Supplementary file 6 — Additional file 6 Table S4 The most enriched (FDR < 0.05) GO terms of season-biased DEGs in the testis of Chinese alligator. [file 12864_2020_7187_MOESM6_ESM.pdf]

Table S4. The most enriched (FDR<0.05) GO terms of season-biased DEGs in the testis of Chinese alligator

GO Accession	Description	Term type	P-Value	Corrected P-Value	DEG number	Background number
**Wild, Winter-biased**						
GO:0005840	ribosome	cellular component	7.57E-26	3.48E-22	55	204
GO:0003735	structural constituent of ribosome	molecular function	9.06E-25	2.08E-21	54	205
GO:0043043	peptide biosynthetic process	biological process	8.11E-24	1.24E-20	69	340
GO:0006518	peptide metabolic process	biological process	1.34E-23	1.40E-20	71	361
GO:0006412	translation	biological process	1.52E-23	1.40E-20	67	328
GO:0043604	amide biosynthetic process	biological process	6.23E-23	4.78E-20	70	360
GO:0043603	cellular amide metabolic process	biological process	4.79E-21	3.15E-18	72	404
GO:0030529	ribonucleoprotein complex	cellular component	2.52E-20	1.45E-17	60	304
GO:0043229	intracellular organelle	cellular component	2.70E-18	1.38E-15	229	2521
GO:0043226	organelle	cellular component	1.49E-17	6.84E-15	231	2591
GO:0010467	gene expression	biological process	3.51E-16	1.47E-13	216	2485
GO:0044424	intracellular part	cellular component	7.28E-16	2.79E-13	263	3185
GO:1901566	organonitrogen compound biosynthetic process	biological process	1.25E-15	4.41E-13	88	685
GO:0044271	cellular nitrogen compound biosynthetic process	biological process	1.97E-15	6.48E-13	207	2374
GO:0005622	intracellular	cellular component	8.76E-15	2.69E-12	277	3498
GO:0044260	cellular macromolecule metabolic process	biological process	1.38E-14	3.98E-12	319	4237
GO:0044267	cellular protein metabolic process	biological process	2.06E-14	5.56E-12	163	1726
GO:0034645	cellular macromolecule biosynthetic process	biological process	2.34E-13	5.97E-11	203	2433
GO:0043232	intracellular non-membrane-bounded organelle	cellular component	7.87E-13	1.90E-10	93	810
GO:0043228	non-membrane-bounded organelle	cellular component	8.39E-13	1.90E-10	95	837
GO:1901576	organic substance biosynthetic process	biological process	8.91E-13	1.90E-10	232	2927
GO:0009059	macromolecule biosynthetic process	biological process	9.09E-13	1.90E-10	203	2467
GO:0044237	cellular metabolic process	biological process	1.04E-12	2.09E-10	361	5128
GO:0044249	cellular biosynthetic process	biological process	1.89E-12	3.62E-10	228	2876
GO:0034641	cellular nitrogen compound metabolic process	biological process	2.96E-12	5.44E-10	267	3530
GO:0005623	cell	cellular component	3.25E-12	5.54E-10	285	3815
GO:0044464	cell part	cellular component	3.25E-12	5.54E-10	285	3815
GO:0009058	biosynthetic process	biological process	3.51E-12	5.76E-10	235	3023
GO:0005198	structural molecule activity	molecular function	1.41E-11	2.24E-09	77	657
GO:1901564	organonitrogen compound metabolic process	biological process	3.37E-11	5.16E-09	98	956
GO:0044444	cytoplasmic part	cellular component	4.54E-11	6.59E-09	105	1059
GO:0043170	macromolecule metabolic process	biological process	4.58E-11	6.59E-09	333	4760
GO:0006807	nitrogen compound metabolic process	biological process	6.73E-11	9.39E-09	271	3698
GO:0044238	primary metabolic process	biological process	8.12E-11	1.09E-08	370	5445
GO:0005634	nucleus	cellular component	8.26E-11	1.09E-08	130	1407
GO:0043231	intracellular membrane-bounded organelle	cellular component	1.54E-10	1.97E-08	168	2003
GO:0043227	membrane-bounded organelle	cellular component	1.66E-10	2.06E-08	168	2005
GO:0032991	macromolecular complex	cellular component	2.73E-10	3.31E-08	158	1852
GO:0005737	cytoplasm	cellular component	4.46E-10	5.26E-08	129	1463
GO:0071704	organic substance metabolic process	biological process	5.75E-10	6.62E-08	378	5671
GO:0019538	protein metabolic process	biological process	2.46E-09	2.76E-07	179	2269
GO:0003676	nucleic acid binding	molecular function	1.34E-07	1.46E-05	197	2715
GO:1901363	heterocyclic compound binding	molecular function	2.46E-07	2.63E-05	292	4337
GO:0097159	organic cyclic compound binding	molecular function	2.52E-07	2.63E-05	292	4338
GO:0008152	metabolic process	biological process	2.48E-06	0.00025333	411	6693
GO:0004719	protein-L-isoaspartate (D-aspartate) O-methyltransferase activity	molecular_function	2.74E-05	0.002736	7	21
GO:0016070	RNA metabolic process	biological process	4.51E-05	0.0044187	160	2281
GO:0032259	methylation	biological process	4.66E-05	0.0044673	13	74
GO:0006139	nucleobase-containing compound metabolic process	biological_process	7.14E-05	0.006707	208	3121
GO:0034654	nucleobase-containing compound biosynthetic process	biological_process	9.21E-05	0.0084762	138	1951
GO:0017025	TBP-class protein binding	molecular function	0.00013706	0.012365	4	7
GO:0006725	cellular aromatic compound metabolic process	biological process	0.00014541	0.012866	215	3275
GO:0008276	protein methyltransferase activity	molecular function	0.00017316	0.014662	10	49
GO:1901360	organic cyclic compound metabolic process	biological process	0.00017786	0.014662	216	3307
GO:0006351	transcription, DNA-templated	biological process	0.00018088	0.014662	126	1783
GO:0097659	nucleic acid-templated transcription	biological process	0.00018088	0.014662	126	1783
GO:0046483	heterocycle metabolic process	biological process	0.00018164	0.014662	214	3268
GO:0090304	nucleic acid metabolic process	biological process	0.00025781	0.020361	191	2891
GO:0019438	aromatic compound biosynthetic process	biological process	0.00026109	0.020361	143	2073
GO:0010340	carboxyl-O-methyltransferase activity	molecular function	0.00030028	0.022528	7	29
GO:0051998	protein carboxyl O-methyltransferase activity	molecular function	0.00030028	0.022528	7	29
GO:0044391	ribosomal subunit	cellular component	0.00030357	0.022528	5	14
GO:0032774	RNA biosynthetic process	biological process	0.00032843	0.023986	126	1807
GO:0018130	heterocycle biosynthetic process	biological process	0.00038405	0.027609	143	2091
GO:1901362	organic cyclic compound biosynthetic process	biological process	0.00044856	0.031751	144	2120
GO:0005845	mRNA cap binding complex	cellular component	0.00058047	0.039862	3	4
GO:0034518	RNA cap binding complex	cellular component	0.00058047	0.039862	3	4
GO:0005739	mitochondrion	cellular component	0.0006458	0.043696	28	276
GO:0000413	protein peptidyl-prolyl isomerization	biological process	0.00070347	0.044954	5	16
GO:0003755	peptidyl-prolyl cis-trans isomerase activity	molecular function	0.00070347	0.044954	5	16
GO:0016859	cis-trans isomerase activity	molecular function	0.00070347	0.044954	5	16
GO:0018208	peptidyl-proline modification	biological process	0.00070347	0.044954	5	16
GO:0018193	peptidyl-amino acid modification	biological process	0.00079025	0.049807	9	48
**Wild, Summer-biased**						
/						
**Warmroom, Winter-biased**						
GO:0003676	nucleic acid binding	molecular function	4.51E-12	2.07E-08	236	2715
GO:0034641	cellular nitrogen compound metabolic process	biological process	2.79E-11	6.42E-08	286	3530
GO:0043229	intracellular organelle	cellular component	8.60E-11	1.32E-07	218	2521

GO:0010467	gene expression	biological process	1.24E-10	1.42E-07	212	2485
GO:0044237	cellular metabolic process	biological process	2.01E-10	1.55E-07	384	5128
GO:0006807	nitrogen compound metabolic process	biological process	2.02E-10	1.55E-07	293	3698
GO:0043226	organelle	cellular component	2.91E-10	1.92E-07	220	2591
GO:0005634	nucleus	cellular component	4.44E-10	2.43E-07	138	1407
GO:0044260	cellular macromolecule metabolic process	biological process	4.75E-10	2.43E-07	328	4237
GO:0005622	intracellular	cellular component	7.94E-10	3.65E-07	278	3498
GO:0043231	intracellular membrane-bounded organelle	cellular component	1.03E-09	4.29E-07	178	2003
GO:0043227	membrane-bounded organelle	cellular component	1.12E-09	4.29E-07	178	2005
GO:0044424	intracellular part	cellular component	2.74E-09	9.71E-07	255	3185
GO:0044271	cellular nitrogen compound biosynthetic process	biological process	4.94E-09	1.62E-06	198	2374
GO:1901576	organic substance biosynthetic process	biological process	9.39E-09	2.88E-06	233	2927
GO:0044249	cellular biosynthetic process	biological process	1.53E-08	4.41E-06	229	2876
GO:0009058	biosynthetic process	biological process	2.99E-08	8.10E-06	236	3023
GO:0071704	organic substance metabolic process	biological process	4.09E-08	1.05E-05	405	5671
GO:1901363	heterocyclic compound binding	molecular function	4.67E-08	1.09E-05	326	4337
GO:0097159	organic cyclic compound binding	molecular function	4.81E-08	1.09E-05	326	4338
GO:0044238	primary metabolic process	biological process	4.96E-08	1.09E-05	391	5445
GO:0043170	macromolecule metabolic process	biological process	7.09E-08	1.42E-05	348	4760
GO:0005623	cell	cellular component	7.42E-08	1.42E-05	287	3815
GO:0044464	cell part	cellular component	7.42E-08	1.42E-05	287	3815
GO:0030529	ribonucleoprotein complex	cellular component	1.05E-07	1.93E-05	40	304
GO:1901360	organic cyclic compound metabolic process	biological process	1.59E-07	2.81E-05	254	3307
GO:0006725	cellular aromatic compound metabolic process	biological process	1.67E-07	2.84E-05	252	3275
GO:0016070	RNA metabolic process	biological process	1.83E-07	2.90E-05	186	2281
GO:0034645	cellular macromolecule biosynthetic process	biological process	1.83E-07	2.90E-05	193	2433
GO:0046483	heterocycle metabolic process	biological process	2.21E-07	3.39E-05	251	3268
GO:0006139	nucleobase-containing compound metabolic process	biological_process	2.29E-07	3.40E-05	241	3121
GO:0005840	ribosome	cellular component	2.41E-07	3.47E-05	30	204
GO:0003735	structural constituent of ribosome	molecular function	2.87E-07	4.00E-05	30	205
GO:0009059	macromolecule biosynthetic process	biological process	3.31E-07	4.48E-05	194	2467
GO:0090304	nucleic acid metabolic process	biological process	5.37E-07	7.06E-05	224	2891
GO:0043043	peptide biosynthetic process	biological process	5.86E-07	7.49E-05	42	340
GO:0006518	peptide metabolic process	biological process	1.27E-06	0.00015562	43	361
GO:0043604	amide biosynthetic process	biological process	1.29E-06	0.00015562	43	360
GO:1901566	organonitrogen compound biosynthetic process	biological process	2.53E-06	0.00029822	69	685
GO:0006412	translation	biological process	2.97E-06	0.00034127	39	328
GO:0043603	cellular amide metabolic process	biological process	6.50E-06	0.00072913	45	404
GO:0008152	metabolic process	biological process	2.71E-05	0.0029686	447	6693
GO:0016071	mRNA metabolic process	biological process	2.95E-05	0.0031616	25	166
GO:0046660	female sex differentiation	biological process	5.73E-05	0.0059929	5	8
GO:0006396	RNA processing	biological process	6.39E-05	0.0065304	45	403
GO:0019438	aromatic compound biosynthetic process	biological process	6.53E-05	0.0065304	160	2073
GO:1901362	organic cyclic compound biosynthetic process	biological process	8.97E-05	0.0087767	162	2120
GO:0018130	heterocycle biosynthetic process	biological process	0.00010548	0.010111	160	2091
GO:0034654	nucleobase-containing compound biosynthetic process	biological_process	0.00011041	0.010367	150	1951
GO:0032259	methylation	biological process	0.00017888	0.01646	13	74
GO:0044428	nuclear part	cellular component	0.00021976	0.019825	48	483
GO:0060255	regulation of macromolecule metabolic process	biological process	0.00029908	0.026463	127	1639
GO:0080090	regulation of primary metabolic process	biological process	0.0003708	0.03219	126	1631
GO:0006351	transcription, DNA-templated	biological process	0.00043278	0.036204	135	1783
GO:0097659	nucleic acid-templated transcription	biological process	0.00043278	0.036204	135	1783
GO:0007548	sex differentiation	biological process	0.0005042	0.041426	5	12
GO:0032774	RNA biosynthetic process	biological process	0.00055953	0.044911	136	1807
GO:0010468	regulation of gene expression	biological process	0.00056615	0.044911	121	1568
GO:0044267	cellular protein metabolic process	biological process	0.00061535	0.047163	134	1726
GO:1901564	organonitrogen compound metabolic process	biological process	0.00061897	0.047163	79	956
GO:0043228	non-membrane-bounded organelle	cellular component	0.00062529	0.047163	71	837
**Warmroom, Summer-biased**						
GO:0005509	calcium ion binding	molecular function	1.53E-06	0.0070614	68	470
